# NanH Is Produced by Sporulating Cultures of Clostridium perfringens Type F Food Poisoning Strains and Enhances the Cytotoxicity of C. perfringens Enterotoxin

**DOI:** 10.1128/mSphere.00176-21

**Published:** 2021-04-28

**Authors:** Jihong Li, Bruce A. McClane

**Affiliations:** a Department of Microbiology and Molecular Genetics, University of Pittsburgh School of Medicine, Pittsburgh, Pennsylvania, USA; University of Kentucky

**Keywords:** *Clostridium perfringens*, enterotoxin, sialidase, NanH, Spo0A, sporulation

## Abstract

Clostridium perfringens type F food poisoning (FP) strains cause one of the most common foodborne illnesses. This FP develops when type F FP strains sporulate in the intestines and produce C. perfringens enterotoxin (CPE), which is responsible for the diarrhea and abdominal cramps of this disease. While C. perfringens can produce up to three different sialidases, the current study surveyed FP strains, which confirmed the results of a previous study that they consistently carry the *nanH* sialidase gene, often as their only sialidase gene. NanH production was found to be associated with sporulating cultures of the surveyed type F FP strains, including SM101 (a transformable derivative of a FP strain). The sporulation-associated regulation of NanH production by strain SM101 growing in modified Duncan-Strong medium (MDS) was shown to involve Spo0A, but it did not require the completion of sporulation. NanH production was not necessary for either the growth or sporulation of SM101 when cultured in MDS. In those MDS cultures, NanH accumulated in the sporulating mother cell until it was released coincidently with CPE. Since CPE becomes extracellular when mother cells lyse to release their mature spores, this indicates that mother cell lysis is also important for NanH release. The copresence of NanH and CPE in supernatants from lysed sporulating cultures was shown to enhance CPE cytotoxicity for Caco-2 cells. This enhancement was attributable to NanH increasing CPE binding and could be replicated with purified recombinant NanH. These *in vitro* findings suggest that NanH may be an accessory virulence factor during type F FP.

**IMPORTANCE** Clostridium perfringens type F strains cause the second most common bacterial foodborne illness in the United States. C. perfringens enterotoxin (CPE) is responsible for the diarrhea and cramping symptoms of this food poisoning (FP). Previous studies showed that NanI sialidase can enhance CPE activity *in vitro*. While many type F FP strains do not produce NanI, they do consistently make NanH sialidase. This study shows that, like CPE, NanH is produced by sporulating type F FP strains and then released extracellularly when their sporulating cells lyse to release their mature spore. NanH was shown to enhance CPE cytotoxicity *in vitro* by increasing CPE binding to cultured Caco-2 cells. This enhancement could be important because many type F FP strains produce less CPE than necessary (in a purified form) to cause intestinal pathology in animal models. Therefore, NanH represents a potential accessory virulence factor for type F FP.

## Abstract (precis)

Clostridium perfringens type F strains cause the second most common bacterial foodborne illness in the United States. C. perfringens enterotoxin (CPE) is responsible for the diarrhea and cramping symptoms of this food poisoning (FP). Previous studies showed that NanI sialidase can enhance CPE activity *in vitro*.

## INTRODUCTION

Clostridium perfringens type F strains cause the second most common bacterial foodborne disease of humans (1). In the United States, C. perfringens type F food poisoning (FP) affects about 1 million people per year, causing annual economic losses of >$310 million (2, 3). This FP is occasionally fatal in the elderly, debilitated, or chronically ill persons (1, 4). The enteric virulence of type F strains is largely attributable to their production of C. perfringens enterotoxin (CPE), a 35-kDa pore-forming toxin that binds to claudin receptors (5, 6). Under certain predisposing conditions, type F enteric infections can further develop into a lethal enterotoxemia when CPE produced in the intestines is absorbed into the blood and then damages organs such as the liver, heart, and kidney (7, 8).

Once ingested in contaminated foods, C. perfringens type F FP strains grow rapidly in the intestines before sporulating (1, 9). This *in vivo* sporulation plays a critical role in type F FP. i.e., CPE is produced only when FP strains sporulate in the intestines (1, 9). Furthermore, this enterotoxin lacks a signal peptide for secretion, so it is released into the intestinal lumen only when the mother cell lyses to free its mature spore (10).

Type F strains producing CPE also cause 5 to 15% of all cases of human nonfoodborne gastrointestinal diseases (NFD), which include antibiotic-associated diarrhea and sporadic diarrhea (1, 11). Cases of CPE-associated NFD are typically more severe than type F FP cases. Those NFD cases are also usually of longer duration (lasting up to several weeks) than type F FP cases, which typically self-resolve within 24 h (1, 11).

C. perfringens is known to produce ∼20 different toxins, but individual strains vary in their toxin production (6, 12, 13). Based upon their carriage of genes encoding six of these toxins (alpha, beta, epsilon, and iota toxins, CPE, and NetB), C. perfringens strains are classified into seven toxinotypes (types A through G) (14). In this classification scheme, type F strains carry the genes encoding alpha toxin and CPE, which can be encoded by either a chromosomal or plasmid-borne gene (*cpe*) (14–18). Most type F FP strains carry a chromosomal *cpe* gene, while type F NFD strains almost always carry a plasmid-borne *cpe* gene (15–17). Except for their partial similarity to some type C strains, type F FP isolates with a chromosomal *cpe* gene are a genetically distinct lineage from most other C. perfringens strains (including type F NFD strains) (18, 19). These genetic differences have phenotypic consequences that may impact virulence. For example, type F FP strains produce more resistant spores than most other C. perfringens strains (9, 19), but unlike most other C. perfringens strains, these FP strains do not produce perfringolysin O as they lack the *pfoA* gene (18).

In addition to their toxins, C. perfringens produces an array of enzymes to attack host tissues. For example, this bacterium can produce up to three different sialidases, named NanJ, NanI, and NanH (20). NanJ and NanI are secreted from C. perfringens but NanH is cytoplasmic, at least in early- to mid-log-phase vegetative cultures (20). Our group recently demonstrated that NanI sialidase potentiates CPE action by increasing the binding of this toxin to cultured Caco-2 cells (21). Furthermore, it was shown that NanI contributes to long-term colonization of the mouse small intestine by C. perfringens NFD strain F4969 (22).

However, like the *pfoA* gene, the *nanI* gene is also absent from the typical type F FP strains that carry a chromosomal *cpe* gene (23). The inability of those FP strains to produce NanI may reduce their ability to colonize the intestines, which could help to explain why these FP strains cause an acute gastrointestinal (GI) disease lasting only 12 to 24 h rather than the GI disease lasting up to several weeks caused by NanI-positive NFD strains (11). Although lacking *nanI*, it has been reported that these type F FP strains often carry the *nanH* (and sometimes also the *nanJ*) sialidase gene (23). The possible contribution of NanH to type F FP strain growth, sporulation, and pathogenicity has not been studied. Therefore, the current study first explored *nanH* gene regulation in, and NanH production by, type F FP strain SM101. A *nanH* null mutant strain was then constructed in strain SM101, and supernatants of that mutant were tested for the ability to induce Caco-2 cell cytotoxicity. In addition, the current study assessed whether purified NanH can affect CPE-induced cytotoxicity.

## RESULTS

### Comparison of sialidase activity among C. perfringens type F FP strains grown in Todd-Hewitt (TH) broth versus modified Duncan-Strong (MDS) sporulation medium.

**FIG 1:**
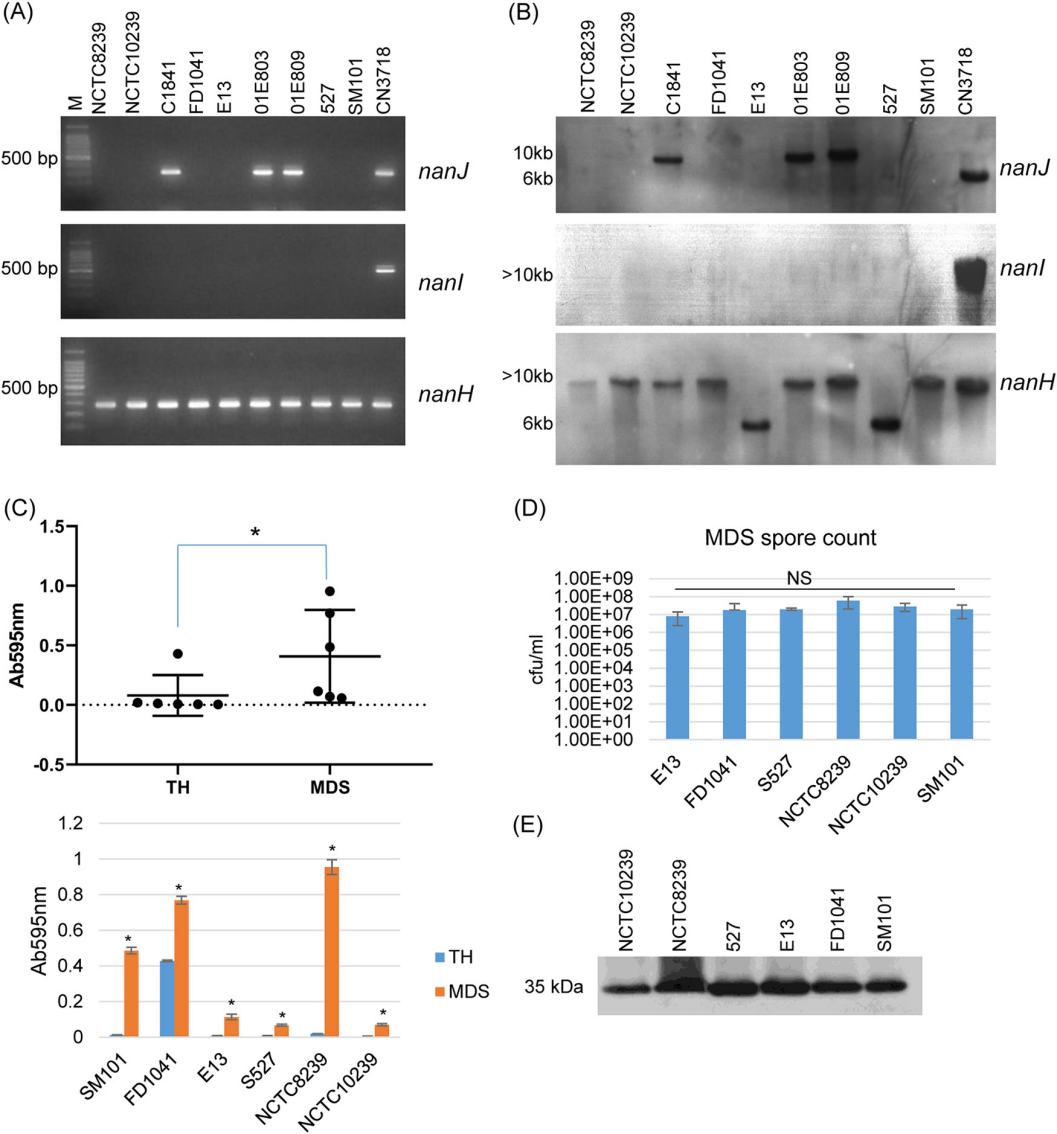
Survey of sialidase gene carriage, supernatant sialidase activity, spore formation, and CPE production for chromosomal *cpe*, type F FP strains. (A) Internal PCR analysis of *nanJ*, *nanI*, and *nanH* gene carriage by type F FP strains (Table 1); DNA from type D strain CN3718, which carries all three sialidase genes, served as a positive control for each PCR. M, 100-bp DNA ladder (purchased from Fisher Scientific). (B) Southern blot analyses of *nanJ*, *nanI*, and *nanH* carriage using DNA from the panel A type F FP strains. DNA from each isolate was digested with BsrGI and hybridized with a *nanJ*-specific probe. After detection and stripping, the same membrane was reprobed with a *nanI*-specific probe. After detection and stripping again, the membrane was reprobed with a *nanH*-specific probe, and hybridization was detected. The size of DNA fragments in kilobases (kb) is shown to the left of the gel. (C) Sialidase activity was measured in supernatants from 24-h TH or MDS culture of type F FP strains that produce only NanH. The top panel shows group sialidase activity comparison for TH versus MDS; the bottom panel shows individual isolate sialidase activity comparison for TH versus MDS. Results shown are the averages of three repetitions; the error bars indicate the standard deviations (SD). *, *P* < 0.05, relative to TH culture supernatant. All data were corrected for background by subtraction of absorbance at 595 nm values for supernatants of 24-h TH or MDS culture mixed with 0.05 M Tris-HCl (pH 7.2) buffer without sialidase substrate. (D) Heat-resistant spore formation levels by the six type F FP strains carrying only the *nanH* gene. The bacteria were grown in MDS for 24 h at 37°C and then heat shocked for 20 min at 70°C. After dilution, the heat-shocked cultures were plated onto BHI agar plates and grown anaerobically overnight at 37°C for colony counting of germinated spores. Results shown are the averages of three repetitions; the error bars indicate the SD. NS, not significantly different (*P* > 0.05). (E) Western blot analyses for CPE production by six type F FP strains using MDS overnight culture supernatants. The size of proteins (in kilodaltons [kDa]) is shown at left. The results shown are representative of three repetitions.

Initial experiments were performed to confirm a previous report (23) regarding sialidase gene carriage by nine chromosomal *cpe*, type F FP strains. For this purpose, PCR and Southern blot analyses were used to survey the presence of each C. perfringens sialidase gene (*nanJ*, *nanI*, and *nanH*) in those type F FP strains. The obtained PCR results (Fig. 1A) indicated that DNA from each of the nine type F FP strains supported amplification of a *nanH* product, with DNA from three strains also supporting amplification of a *nanJ* gene product. DNA from these nine FP strains did not support amplification of a *nanI* gene product. Southern blot analyses (Fig. 1B) using *nanH*, *nanI*- and *nanJ-*specific probes were fully consistent with the PCR amplification results.

Sialidase activity was then assessed in culture supernatants of the six type F FP strains carrying the *nanH* gene, but not the *nanJ* or *nanI* gene. Only one of these strains had detectible sialidase activity in supernatants of 24-h TH cultures (Fig. 1C). However, when these six type F FP strains were grown 24 h in MDS sporulation medium, each of their culture supernatants had significantly higher sialidase activity compared to that in their TH culture supernatants and overall sialidase activity was significantly higher between MDS versus TH cultures of these FP strains (Fig. 1C).

Those six FP strains all sporulated well in MDS, producing similarly large amounts of heat-resistant spores (Fig. 1D). Consistent with that finding and the fact that CPE production is sporulation dependent (10), these FP strains also produced similar levels of CPE in MDS (Fig. 1E). Notably, the five strains that did not produce sialidase in TH medium, which is generally considered a vegetative growth medium for C. perfringens, also sporulated poorly (<10 spores per ml) and produced little or no CPE in that medium after 24 h (not shown). Interestingly, the one strain (FD1041) producing NanH in TH medium also produced ∼10^4^ heat-resistant spores and sizeable amounts of CPE when grown 24 h in that medium.

**FIG 2:**
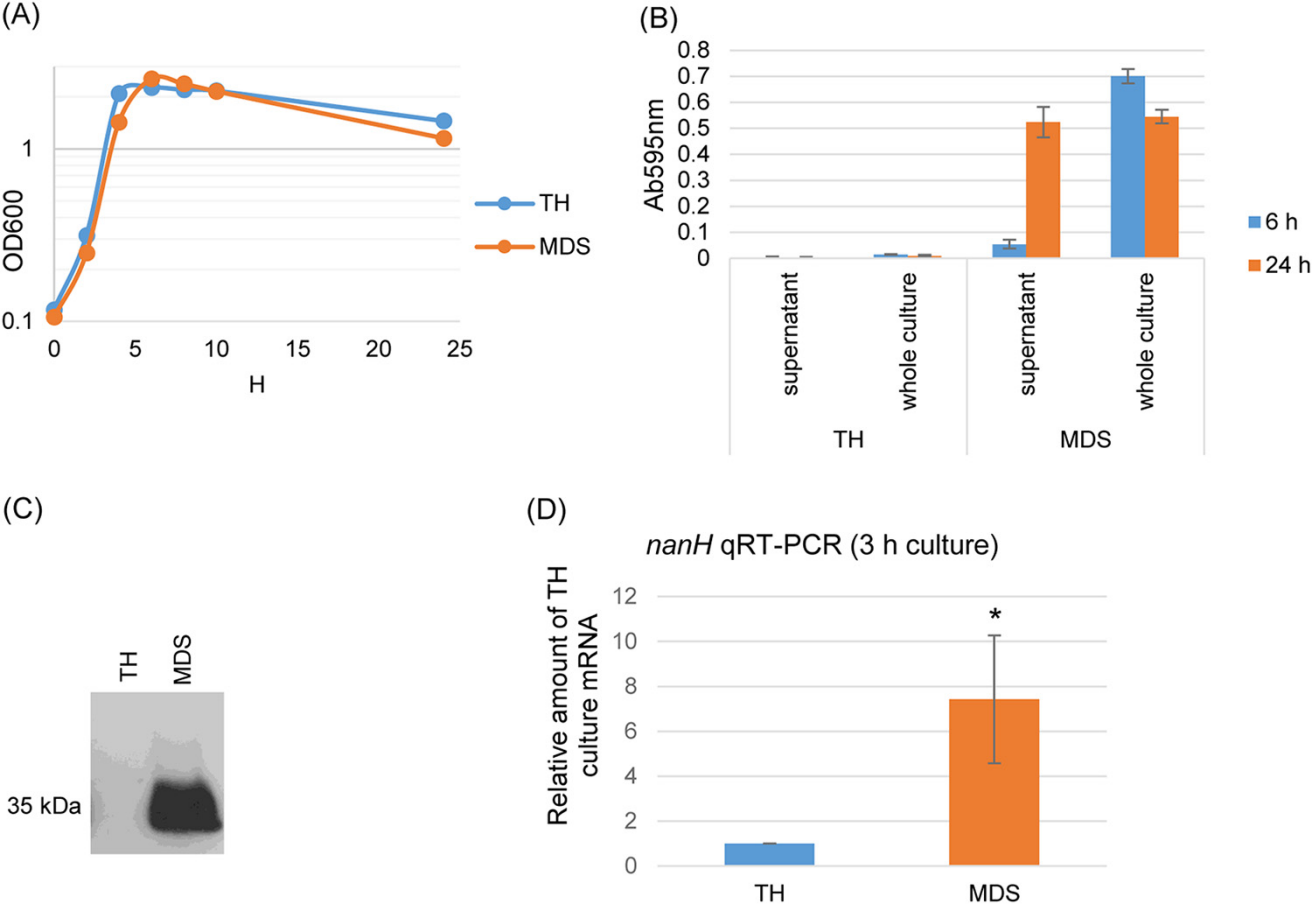
Comparison of growth, sialidase activity, CPE production, and NanH production when strain SM101 is cultured in TH versus MDS media. (A) Postinoculation changes in the OD_600_ for cultures of wild-type strain SM101 growing in TH or MDS at 37°C. Every 2 h up to 10 h of culture, and then again at 24 h, a 1-ml aliquot of the culture was removed, and the OD_600_ was determined. Time (in hours) is shown on the *x* axis. Results shown are representative of three repetitions. (B) SM101 sialidase activity measured for 6 h or 24 h in TH and MDS cultures using culture supernatants (which contain only extracellular NanH) or sonicated whole cultures (which contain both intracellular and extracellular NanH). Results shown are the averages of three repetitions; the error bars indicate the SD. (C) SM101 CPE Western blot analyses using supernatants from 24-h TH and MDS cultures. The size of proteins in kilodaltons is shown to the left of the blot. The result shown is representative of three repetitions. (D) qRT-PCR analyses of *nanH* transcription were performed using 20 ng of SM101 RNA isolated from TH or MDS cultures. Average threshold cycle (*C_T_*) values were normalized to the value for the housekeeping 16S RNA gene, and the fold differences were calculated using the comparative *C_T_* (2^−ΔΔCT^) method. The value of each bar indicates the calculated MDS culture fold change relative to the value for the TH culture. Shown are the mean values from three independent experiments; the error bars indicate the S.D. *, *P* < 0.05 relative to TH culture supernatant.

To further investigate the possible association between NanH production and sporulation, we focused on type F FP strain SM101 since this strain is a transformable derivative of a chromosomal *cpe* type F FP strain (24). To initiate this work, growth curves were compared for strain SM101 cultured in either TH medium or MDS, with the results shown in Fig. 2A. This experiment demonstrated that this strain grows well in both media, with only small differences.

Since NanH lacks a signal peptide for active secretion (24), sialidase activity was measured at two time points (6 h or 24 h) for TH and MDS SM101 cultures, using culture supernatants (which contain only extracellular NanH) and sonicated whole cultures (which contain both intracellular and extracellular NanH). The results (Fig. 2B) indicated that, in TH medium, sialidase activity was negligible, even after 24 h, in both culture supernatants and sonicated whole cultures. However, for MDS cultures, substantial sialidase activity (absorbance at 595 nm [Ab_595_]) was detected in both 6-h and 24-h sonicated whole cultures. In contrast, only a small amount of sialidase activity was detected in 6-h MDS culture supernatant, although by 24 h (a time when most cells in the culture have sporulated and lysed, as evident from the mother cell lysis-dependent release of CPE), substantial sialidase activity was present in MDS culture supernatants (Fig. 2C). A *nanH* quantitative reverse transcriptase PCR (qRT-PCR) was also performed to compare *nanH* expression levels between 3-h TH and MDS cultures of SM101. Results of that analysis showed that, compared to TH cultures, *nanH* expression significantly increases in MDS cultures (Fig. 2D).

**FIG 3:**
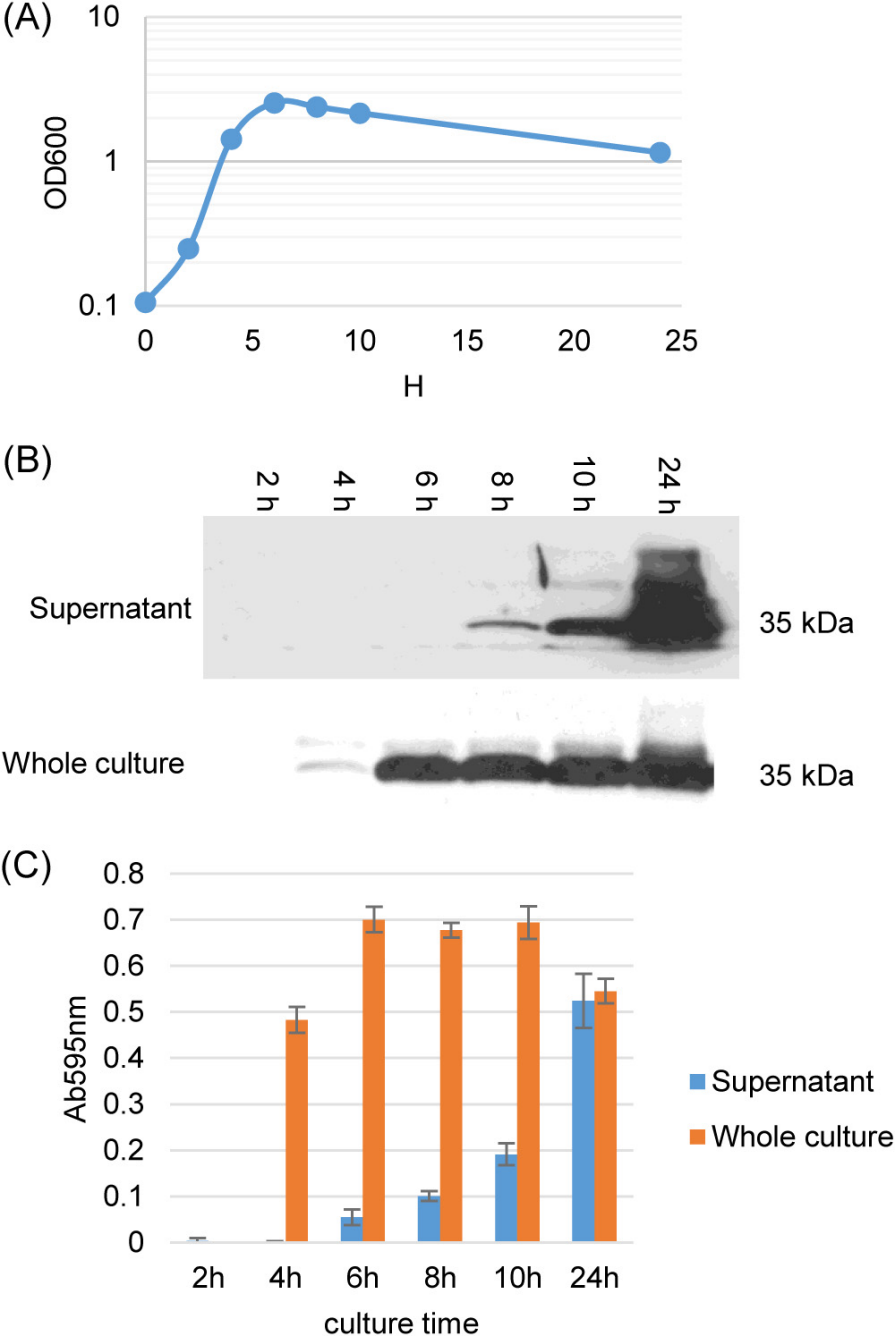
Kinetics of growth, sialidase activity, and CPE production when strain SM101 is cultured in MDS. (A) Postinoculation changes in the OD_600_ for wild-type SM101 cultured in MDS sporulation medium at 37°C. Every 2 h up to 10 h, and then again at 24 h, a 1-ml aliquot of the culture was removed, and the OD_600_ was determined. Results shown are representative of three repetitions. (B) CPE Western blot analyses of MDS culture supernatants and sonicated whole cultures from every 2 h of culture up to 10 h, and then again at 24 h. Results shown are representative of three repetitions. The size of protein (in kilodaltons) is shown to the right of the blot. (C) Sialidase activity analyses using MDS culture supernatant and whole culture from every 2 h of culture up to 10 h, and then at 24 h. Shown are the mean values from three independent experiments. The error bars indicate the SD.

Since Fig. 2 results indicated that NanH, like CPE, is predominantly produced by strain SM101 in sporulating cultures, the kinetics of NanH and CPE production and extracellular release for MDS cultures of this strain were more closely examined (Fig. 3). With consideration of the SM101 growth curve in MDS (Fig. 3A), CPE production (Fig. 3B) began in late log-stationary phase (i.e., by ∼4 to 6 h) in sporulating MDS SM101 cultures. In comparison, the presence of CPE in supernatants of those cultures was detectable more slowly (by 8 h) and then became substantial between 10 and 24 h. That result is consistent with intracellular CPE being released when mother cells lyse to release their endospore (10), an effect that begins after 8 h when SM101 is cultured in MDS medium at 37°C (not shown).

For strain SM101, sialidase activity is attributable to NanH production since this is the only sialidase made by this strain based upon Fig. 1 PCR and Southern results (and phenotypically confirmed later in Fig. S2C in the supplemental material using an SM101 *nanH* mutant). When sialidase activity due to NanH was measured in the same samples used to assess CPE production in Fig. 3B, this analysis revealed that NanH is produced earlier than CPE in sporulating MDS cultures and then plateaus after 6 h (Fig. 3C). Similar to CPE release, most extracellular NanH activity appeared between 10 and 24 h in SM101 MDS sporulating cultures. Collectively, these results strongly suggest that NanH resembles CPE, which also lacks a signal peptide for secretion (25), in being produced by sporulating FP MDS cultures, accumulating intracellularly, and then being released extracellularly when the mother cell lyses.

10.1128/mSphere.00176-21.2 FIG S2 Intron-based mutagenesis to create an SM101 *nanH* null mutant and characterization of that mutant and a complementing strain when cultured in MDS medium. (A) *nanH* internal PCR analysis for wild-type SM101, its *nanH* null mutant, and a *nanH* complementing strain. Without an intron insertion, the *nanH* PCR product amplified from wild-type SM101 was ∼314 bp. With an ∼900-bp intron insertion, the PCR product amplified for the *nanH* null mutant strain was ∼1,200 bp using the same primers. The complementing strain, which has the wild-type *nanH* gene, amplified a PCR product of the same size as wild-type SM101. The size of DNA is shown to the left. (B) Southern blot analysis of intron-specific probe hybridization with DNA from wild-type SM101 or its *nanH* null mutant. DNA from both strains was digested with EcoRI and electrophoresed on a 1% agarose gel prior to blotting and hybridization with an intron-specific probe. The size of DNA fragments (in kilobases) is shown to the right. (C) Postinoculation changes in OD_600_ for cultures of wild-type SM101, its *nanH* null mutant, and the complementing strain cultured in MDS at 37°C. Every 2 h of culture up to 10 h, and then again at 24 h, a 1-ml aliquot of the culture was removed, and the OD_600_ was determined. Shown is a representative growth curve of three repetitions. (D) Sialidase activity analyses for wild-type, *nanH* null mutant, or *nanH* complementing strains using 6-h or 24-h MDS culture supernatants or sonicated MDS whole culture. *, *P* < 0.05 relative to the wild type. (E) Western blot analyses for CPE production by wild-type SM101, the *nanH* null mutant, or a *nanH* complementing strain using 6-h sonicated whole MDS culture (upper panel) or 24-h MDS culture supernatants or sonicated MDS whole culture (lower panel). Shown is a representative Western blot for three repetitions. The size of proteins (in kilodaltons) is shown to the left. (F) Numbers of vegetative cells or heat-resistant spores made by SM101, its *nanH* null mutant, or the *nanH* complementing strain when cultured in MDS for 24 h at 37°C. After this incubation, half of the culture was 10-fold serially diluted with distilled water, heat shocked for 20 min at 70°C, and plated onto BHI agar plates. Those plates were then grown anaerobically overnight at 37°C for colony counting of germinated spores. The other half of each culture was diluted and plated, without heat shocking, onto BHI agar plates (to calculate vegetative cells). After overnight anaerobic incubation at 37°C, colony counting was performed. Results in panels D and F are the averages of three repetitions; the error bars indicate the S.D. All heat-resistant spores measured between strains were not significant (*P* > 0.05). Download FIG S2, TIF file, 1.7 MB. Copyright © 2021 Li and McClane. 2021 Li and McClane. https://creativecommons.org/licenses/by/4.0/ This content is distributed under the terms of the Creative Commons Attribution 4.0 International license.

### Spo0A is a major regulator of NanH production in MDS sporulating cultures of SM101.

To help explain why MDS sporulating cultures of strain SM101 so strongly produce NanH, the motif alignment & search tool (MAST) (http://meme-suite.org/tools/mast) was used to analyze sequences upstream of the SM101 *nanH* open reading frame (ORF) (24) for the presence of binding motifs for sporulation-associated regulators. This analysis identified the presence of two binding motifs for Spo0A (which is required to initiate C. perfringens sporulation [26]) about 231 bp and 297 bp and two binding motifs for SigE (which is a sporulation-associated sigma factor) about 404 bp and 450 bp, respectively, upstream of the *nanH* start codon.

To test whether SigE is involved in NanH production, the *sigE* gene was inactivated in strain SM101 using *Clostridium*-modified Targetron technology (27), creating a *sigE* null mutant named SM101sigEKO (KO stands for knockout). PCR and Southern blot analyses confirmed that an intron had inserted into the *sigE* gene of this mutant and that its genome contained only a single intron insertion (Fig. S1A and B). A complementing strain named SM101sigEc was also prepared, which was confirmed by PCR showing that this strain contains a wild-type *sigE* gene (Fig. S1A). Consistent with previous results (28), the SM101 *sigE* null mutant did not make either spores or CPE in MDS medium, while the complementing strain produced nearly the same amounts of spores and CPE as did wild-type SM101 (Fig. S1C and D). When sialidase activity was measured in 24-h MDS culture supernatants, no significant differences in sialidase activity were detected between wild-type SM101, its *sigE* null mutant, or the complementing strain (Fig. S1E).

10.1128/mSphere.00176-21.1 FIG S1 Intron-based mutagenesis to create an SM101 *sigE* null mutant and characterization of that mutant and a complementing strain when cultured in MDS medium. (A) *sigE* internal PCR analysis for wild-type, *sigE* null mutant, and *sigE* complementing strains. Without intron insertion, the *sigE* PCR product amplified from wild-type SM101 was ∼230 bp. With an ∼900-bp intron insertion *in sigE*, the PCR product for *sigE* null mutant strain was 1,130 bp using the same primers. The complementing strain, with a wild-type *sigE* gene, supported PCR amplification of the small band as the wild-type strain. The size of DNA is shown to the left. (B) Southern blot analysis of an intron-specific probe with DNA from wild-type or the *sigE* null mutant strain. DNA from both strains was digested with EcoRI and electrophoresed on a 1% agarose gel prior to blotting and hybridization with an intron-specific probe. The size of DNA fragments, in kilobases, is shown to the right. (C) Heat-resistant spore formation levels for strain SM101, its *sigE* null mutant strain, or the *sigE* complementing strain. The bacteria were grown in MDS for 24 h at 37°C and then heat shocked for 20 min at 70°C. After a 10-fold serial dilution with distilled water, the heat-shocked cultures were plated onto BHI agar plates and grown anaerobically overnight at 37°C for colony counting of germinated spores. *, *P* < 0.05 relative to the wild type. (D) Western blot analyses of CPE production by wild-type SM101, its *sigE* null mutant, or its *sigE* complementing strain using MDS 24-h culture supernatants. The size of proteins (in kilodaltons) is shown to the right. Shown is a representative blot of three repetitions. (E) Sialidase activity analyses for wild-type, *sigE* null mutant, or *sigE* complementing strains using overnight or 24-h MDS culture supernatants. Results shown in panels C and E are the averages of three repetitions; the error bars indicate the standard deviations (S.D.). Sialidase activity differences measured between strains were not significant (*P* > 0.05). Download FIG S1, TIF file, 0.8 MB. Copyright © 2021 Li and McClane. 2021 Li and McClane. https://creativecommons.org/licenses/by/4.0/ This content is distributed under the terms of the Creative Commons Attribution 4.0 International license.

**FIG 4:**
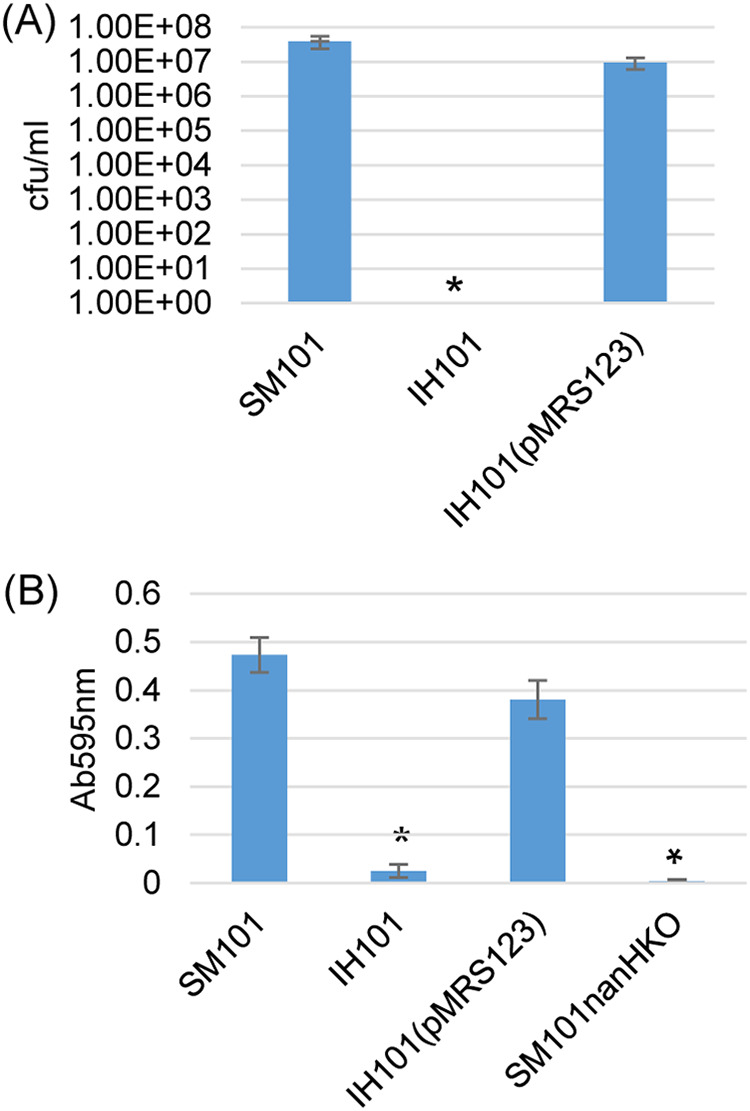
Comparison of spore formation levels and sialidase activity for MDS cultures of SM101 *spo0A* null mutant (IH101) and the complementing strain IH101(pMRS123). (A) Heat-resistant spore formation by strain SM101, its *spo0A* null mutant, and a complementing strain. The bacteria were grown 24 h in MDS at 37°C and then heat shocked for 20 min at 70°C. After a 10-fold serial dilution with distilled water, the heat-shocked cultures were plated onto BHI agar plates and grown anaerobically overnight at 37°C for colony counting of germinated spores. Results show the averages of three repetitions; the error bars indicate the SD. *, *P* < 0.05 relative to wild type. (B) Sialidase activity in supernatants from 24-h MDS cultures of the SM101 wild type, its *spo0A* null mutant, and a complementing strain. Results shown are the means of three repetitions; the error bars indicate the SD. *, *P* < 0.05 relative to wild type. Sialidase values shown are after subtraction of values measured in supernatants of 24-h MDS culture with 0.05 M Tris-HCl (pH 7.2) buffer but no sialidase substrate. SM101nanHKO served as a negative control. There was no statistically significant difference in sialidase activity between strains IH101 and SM101nanHKO.

To test whether Spo0A regulates NanH production in MDS cultures of strain SM101, a similar experiment was performed using a previously constructed (26) SM101 *spo0A* null mutant and complementing strain. Compared to wild-type SM101 and the complementing strain, the *spo0A* null mutant was unable to make spores or CPE when cultured in MDS, similar to previous reports that this mutant cannot sporulate or produce CPE when cultured in Duncan-Strong sporulation medium (26). This mutant also produced barely detectable levels of sialidase activity when cultured in MDS (Fig. 4). These results indicate that Spo0A, but not SigE, is a major regulator of *nanH* gene expression when SM101 is grown in MDS.

### Construction and characterization of SM101 *nanH* null mutant and complementary strains.

The Fig. 1 and 3 results indicated that both NanH and CPE are produced by MDS sporulating cultures of FP strains like SM101 and that these two proteins become copresent extracellularly in older MDS sporulating cultures of those strains. Therefore, experiments were performed to test whether NanH affects CPE production levels, spore formation levels, and CPE activity in MDS cultures of strain SM101. For this purpose, an SM101 *nanH* null mutant (named SM101*nanH*KO) was constructed. PCR analyses confirmed that an ∼900-bp intron had inserted into the *nanH* gene of the SM101*nanH*KO (Fig. S2A), i.e., using the same primer set, DNA from the *nanH* null mutant supported amplification of a larger-size PCR product compared to the DNA from the wild-type and complementing strains. An intron-specific Southern blot analysis was also performed to evaluate whether only a single intron had inserted into the genome of the mutant strain. The results (Fig. S2B) showed the absence of intron probe hybridization to wild-type DNA and that only a single intron insertion was present in strain SM101*nanH*KO. A NanH complementing strain (named SM101*nanHc*) was then prepared by transformation of SM101*nanH*KO with a shuttle plasmid carrying the wild-type *nanH* gene.

Since results of the Fig. 1 and 3 experiments indicated that NanH production is associated with sporulating cultures, the growth rates in MDS were compared for wild-type SM101, its *nanH* null mutant, and the *nanH* complementing strain. The results (Fig. S2C) showed that, in MDS, all three strains exhibited similar growth rates. Sialidase activity was also measured in 6-h or 24-h MDS culture supernatants or sonicated whole cultures of those strains. The results (Fig. S2D) indicated that, in the MDS cultures or culture supernatants, all sialidase activity of strain SM101 is attributable to NanH since no sialidase activity was detectable in 6-h or 24-h MDS culture supernatant or sonicated MDS culture supernatants of its *nanH* mutant and sialidase activity was recovered in the SM101 *nanH* complementing strain.

When the same samples were subjected to CPE Western blot analysis, the results (Fig. S2E) showed that CPE production levels were very similar among wild-type SM101, its *nanH* mutant, and the complementing strain (Fig. S2E). Spore formation levels were also similar among these strains (Fig. S2F). Collectively, these results indicated that NanH production does not affect the levels of growth, CPE production, or spore formation under these experimental conditions.

### Construction and characterization of SM101 *cpe* and *cpe nanH* null mutants.

In order to study whether NanH affects CPE action in the presence of other C. perfringens sporulating culture factors, two additional SM101 mutants were prepared. For this purpose, introns were inserted into the *cpe* gene of SM101 or the SM101 *nanH* null mutant to create, respectively, a *cpe* null mutant and a *cpe nanH* double null mutant. PCR (Fig. S3A) and Southern blot analyses (Fig. S3B) verified these two mutants, i.e., the *cpe*, or both the *cpe* and *nanH* genes, had been disrupted by intron insertions, and the *cpe* mutant has a single intron insertion, while the double mutant has two intron insertions.

10.1128/mSphere.00176-21.3 FIG S3 Intron-based mutagenesis to create an SM101 *cpe* null mutant and an SM101 *cpe nanH* double null mutant. (A) *nanH* and *cpe* internal PCR analyses of wild-type SM101, its *cpe* null mutant, its *nanH cpe* double null mutant (SM101DKO), and SM101DKO *nanH* complementing strain (DKOnanHc). In the absence of an intron insertion into its *nanH* or *cpe* gene, the PCR product amplified from wild-type strain using internal *nanH* primers was ∼314 bp (upper panel), and the PCR product was 233 bp (lower panel) using internal *cpe* primers. Consistent with insertion of an ∼900-bp intron insertion, the same *nanH* primers amplified an ∼1,200-bp PCR product for the *nanH* null mutants and the same *cpe* primers amplified a PCR product of ∼1,100 bp for *cpe* null mutants. As expected, *nanH* complementation of the double null mutant restored the presence of the wild-type *nanH* gene, so the *nanH* PCR product amplified was 314 bp. The size of DNA is shown to the left. (B) Southern blot analysis of intron-specific probe hybridization with DNA from wild-type SM101, its *nanH* null mutant, its *cpe* null mutant, and its *nanH cpe* double null mutant. DNA from all strains was digested with EcoRI and, after electrophoresis, blotted and then hybridized with an intron-specific probe. The sizes of DNA fragments (in kilobases) are shown to the right. (C) Heat-resistant spore formation levels by SM101, its *cpe* null mutant strain, its *cpe nanH* double null mutant, and the double null mutant strain complemented to restore the presence of *nanH*. The bacteria were grown 24 h in MDS at 37°C and then heat shocked for 20 min at 70°C. After a 10-fold serial dilution with distilled water, the heat-shocked cultures were plated onto BHI agar plates and grown anaerobically overnight at 37°C for colony counting of germinated spores. Heat-resistant spore formation levels measured between all strains were not significantly different (*P* > 0.05). (D) Western blot analyses (upper panel) of CPE production by wild-type SM101, its *cpe* null mutant, its *nanH cpe* double null mutant, and *nanH* complementing strains using 24-h MDS culture supernatants. The size of proteins (in kilodaltons) is shown to the left. This blot is a representative result of three repetitions. For all repetitions, a duplicate gel was loaded and stained with Coomassie blue to ensure that all samples contained similar total protein (lower panel). (E) Sialidase activity analyses for wild-type SM101, its *cpe* null mutant, its *cpe nanH* double null mutant, and *nanH* complementing strains using 24-h MDS culture supernatants. Results shown in panels C and E are the means of three repetitions; the error bars indicate the S.D. *, *P* < 0.05 relative to wild-type SM101. Download FIG S3, TIF file, 2.6 MB. Copyright © 2021 Li and McClane. 2021 Li and McClane. https://creativecommons.org/licenses/by/4.0/ This content is distributed under the terms of the Creative Commons Attribution 4.0 International license.

When these two mutants were phenotyped, they produced similar numbers of heat-resistant spores as wild-type SM101 after culture for 24 h in MDS medium (Fig. S3C). As expected, neither mutant made Western blot-detectable amounts of CPE under these culture conditions, even using the same amount of 24-h MDS supernatant sample that allowed ready detection of CPE production by strain SM101 (Fig. S3D). The same supernatants of overnight MDS cultures that were used for CPE Western blot analysis were also assayed for their sialidase activity. Results (Fig. S3E) showed that the 24-h MDS culture supernatants of the *cpe* null mutant contained the same amount of NanH activity as those of wild-type SM101, but the matching culture supernatants of the *cpe nanH* double null mutant had no detectable sialidase activity.

We wanted to compare the cytotoxic effects of various 24-h MDS culture supernatants, but sterile MDS was found to contain small molecules that interfered with the accuracy of the lactate dehydrogenase (LDH) cytotoxicity detection kit (data not shown). To overcome this problem, those MDS culture supernatants were buffer exchanged using Thermo Scientific Zeba Spin Desalting Columns, which removed all LDH cytotoxicity kit interference by sterile MDS. In order to confirm that this column did not affect CPE concentrations or sialidase activity, sialidase activity and CPE levels were compared in 24-h MDS culture supernatants before and after buffer exchange. The results demonstrated that neither sialidase activity nor CPE production were significantly changed by buffer exchange (Fig. S4).

10.1128/mSphere.00176-21.4 FIG S4 Comparison of supernatant sialidase activity and CPE levels between strain SM101 and its derivatives before (Bf) and after (Af) buffer exchange. (A) Sialidase activity of 24-h MDS culture supernatants for SM101, an SM101 *nanH* null mutant, a *nanH* complementing strain of that mutant, an SM101 *cpe* null mutant, and an SM101 *cpe nanH* double null mutant before (Bf) and after (Af) HBSS buffer exchange. All results show the averages of three repetitions; the error bars indicate the S.D. Sialidase activity measured between before (Bf) and after (Af) buffer exchange were not significant (*P* > 0.05). (B) Western blot analyses (upper panel) for CPE levels in the same samples used for the panel A sialidase activity assay. The size of proteins (in kilodaltons) is shown to the left. This blot displays a representative result of three experiments. For all repetitions, a duplicate gel was loaded and stained with Coomassie blue to ensure that all samples contained similar protein amounts (lower panel). Download FIG S4, TIF file, 1.7 MB. Copyright © 2021 Li and McClane. 2021 Li and McClane. https://creativecommons.org/licenses/by/4.0/ This content is distributed under the terms of the Creative Commons Attribution 4.0 International license.

### The presence of NanH in MDS culture supernatants enhances CPE binding to, and cytotoxicity in, Caco-2 cells.

Experiments then evaluated whether NanH can enhance CPE cytotoxicity for Caco-2 cells at natural NanH production levels and in a natural background containing other C. perfringens sporulation-associated factors. For this purpose, buffer-exchanged, 24-h MDS culture supernatants of wild-type SM101, the SM101 *nanH*KO null mutant, and the complementing strain were used, each of which contains very similar amounts of CPE (Fig. S2E). For this experiment, HBSS buffer was used as a negative control and buffer-exchanged, 24-h MDS culture supernatants from both the *cpe* null mutant and *cpe nanH* double null mutant were used to assess the involvement of CPE in any measured cytotoxicity.

**FIG 5:**
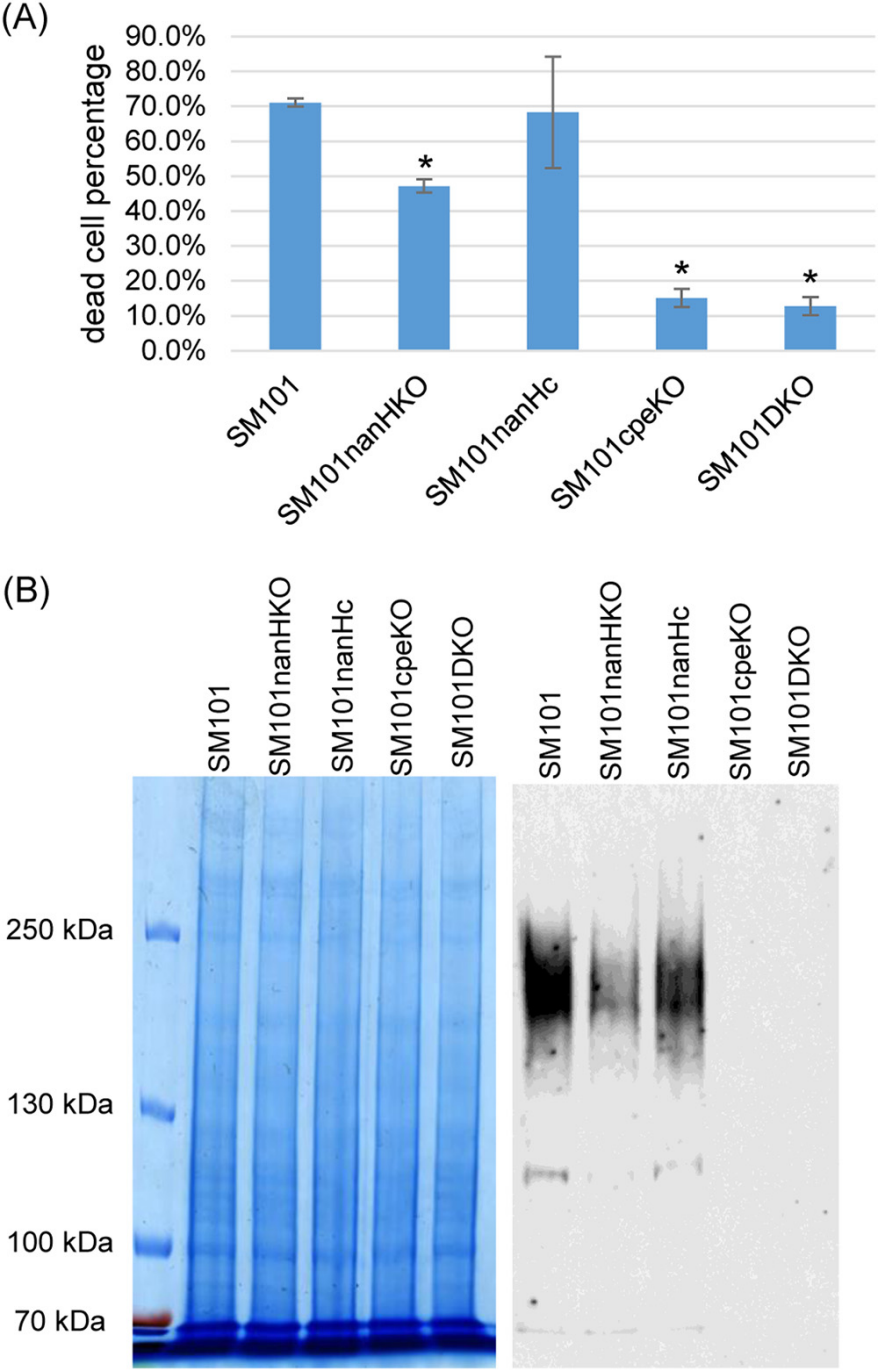
Supernatants containing NanH increase CPE-induced cytotoxicity and CH-1 large complex formation for Caco-2 cells. (A) Percentage of dead Caco-2 cells after treatment at 37°C for 1 h with 24-h MDS culture supernatants (buffer exchanged to HBSS; see Materials and Methods) from CPE-producing wild-type SM101, its *nanH* null mutant, and a *nanH* complementing strain. The effects of similar supernatants from an SM101 *cpe* null mutant and *cpe nanH* double mutant are shown for comparison to ensure CPE involvement in supernatant effects. Shown are the mean values from three independent experiments; the error bars indicate the SD. *, *P* < 0.05 relative to the effects of wild-type supernatants. (B) CPE CH-1 complex formation in Caco-2 cells. Caco-2 cells were treated with 24-h MDS culture supernatant of SM101, its *nanH* null mutant, and a *nanH* complementing strain (all supernatants were buffer exchanged to HBSS; see Materials and Methods and Methods) for 1 h at 37°C. Following incubation, cells were collected and lysed in RIPA buffer, total proteins were separated by SDS-polyacrylamide gel electrophoresis (SDS-PAGE), and CPE Western blotting was performed (right). This blot displays a representative result of three experiments and, to ensure equal levels of proteins in all samples, a duplicate gel was loaded with the same sample, electrophoresed, and stained with Coomassie blue (left). The size of protein is shown to the left of the gel.

As shown in Fig. 5A, MDS supernatants of the *nanH* null mutant strain caused significantly less cytotoxicity than did the MDS supernatants of the wild-type or NanH complementing strain, indicating that NanH contributes to the cytotoxic properties of these supernatants. Confirming major involvement of CPE in the cytotoxic properties of MDS culture supernatants of strain SM101, the MDS culture supernatants of the *cpe* null mutant or *cpe nanH* double null mutant also caused significantly less cytotoxicity compared to MDS culture supernatant of strain SM101.

Since the CH-1 pore complex is required for CPE to cause cytotoxicity in Caco-2 cells, the presence of the CH-1 pore complex in Caco-2 cells was assessed after those cells were treated with the same buffer-exchanged 24-h MDS culture supernatants used in Fig. 5A or after treatment with HBSS, as a negative control. In Caco-2 cells treated with those MDS culture supernatants for 1 h, CPE Western blotting detected less CH-1 pore complex formation in Caco-2 cells treated with MDS supernatants of the *nanH* null mutant compared to equivalent MDS supernatants of the wild-type and complementing strains. As expected, no CH-1 pore complex was formed by treatment with HBSS or with MDS culture supernatants of the *cpe* null mutant or *cpe nanH* null mutant, consistent with the absence of CPE in these two MDS culture supernatants (Fig. 5B).

**FIG 6:**
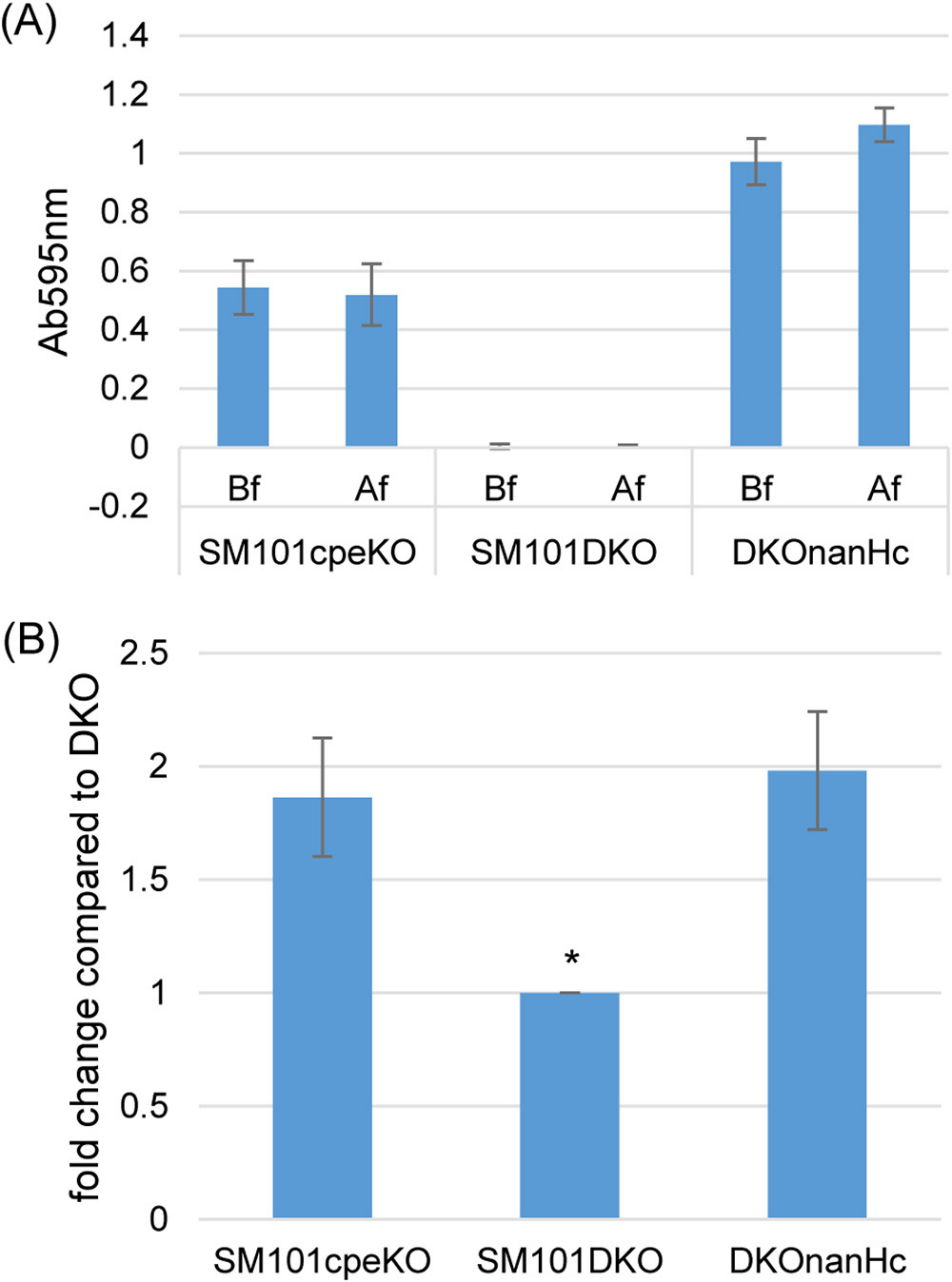
Supernatants containing NanH increase CPE binding to Caco-2 cells. (A) Comparison of supernatant sialidase activity of SM101 *cpe* null mutant, *cpe nanH* double null mutant, and *nanH* complementing to double null mutant. These supernatants were prepared from 24-h MDS cultures, before (Bf) or after (Af) HBSS buffer exchange (see Materials and Methods). There were no significant differences (*P* > 0.05) in sample sialidase activities before or after buffer exchange. (B) Effects of supernatants on AF488-CPE_D48A_ binding to Caco-2 cells. AF488-CPE_D48A_ (5 mg/liter) was added to 24-h supernatants of MDS cultures of SM101cpeko, SM101DKO, and DKOnanHc (after HBSS buffer exchange). These mixes were then applied to Caco-2 cells; after 1 h of incubation at 37°C, the cells were washed three times with HBSS buffer, cells were collected and lysed in 200 μl RIPA buffer, and a 100-μl aliquot of supernatant was read by fluorescence at excitation/emission wavelengths of 485/519 nm. All data shown are corrected for background fluorescence (subtraction of fluorescence in identical samples except for no AF488-CPE_D48A_ addition to the matching buffer-exchanged supernatant). The value of each bar indicates the calculated fold change in AF488-CPE_D48A_ binding for SM101cpeKO and DKOnanHc supernatants relative to the value for AF488-CPE_D48A_ binding to SM101DKO. Shown are the mean values from three independent experiments; the error bars indicate the SD. *, *P* < 0.05 relative to *cpe* null mutant strain.

An experiment was then performed to assess whether the NanH-mediated differences observed in CH-1 pore complex formation were due to NanH affecting the levels of CPE binding to Caco-2 cells. For this experiment, Alexa Fluor 488 (AF488)-labeled rCPE_D48A_ (a recombinant CPE variant that binds to Caco-2 cells similar to CPE but is not cytotoxic because it cannot oligomerize or form the CH-1 pore complex [29]) was added to buffer-exchanged supernatants from MDS cultures of the *cpe* null mutant, *cpe nanH* double null mutant, or the double mutant complemented to produce NanH. Before labeling with AF488, rCPE_D48A_ was purified, quantified, and confirmed by CPE Western blotting (Fig. S5A). A cytotoxicity assay also confirmed that this attenuated CPE variant did not kill Caco-2 cells (Fig. S5B). The same amount (5 μg/ml) of AF488-labeled rCPE_D48A_ (AF488-rCPE_D48A_) was added to buffer-exchanged MDS culture supernatants of the *cpe* null mutant, *cpe nanH* double null mutant, or that same double mutant complemented to produce NanH. No differences were detected between sialidase activity of these samples before (Bf) or after (Af) buffer exchange (Fig. 6A). When these mixtures were added to Caco-2 cells, the fluorescence readings showed that the presence of NanH significantly increased AF488-rCPE_D48A_ binding, i.e., after buffer exchange, the double mutant MDS supernatant supported less AF488-rCPE_D48A_ binding to Caco-2 cells compared to the MDS supernatant of the *cpe* null mutant or the double mutant complemented to produce NanH (Fig. 6B).

10.1128/mSphere.00176-21.5 FIG S5 Production and purification of rCPE_D48A_. (A) Purified rCPE_D48A_ was subjected to SDS-PAGE and then stained with Coomassie blue (left) or Western blotted for CPE (right). (B) Cytotoxicity (percentage of dead Caco-2 cells) after treatment of those cells at 37°C for 1 h with 5 mg/liter of CPE or rCPE_D48A_. All experiments were performed in triplicate, and the mean results are shown. Error bars show S.D. *, *P* < 0.05 relative to native CPE treated. Download FIG S5, TIF file, 0.6 MB. Copyright © 2021 Li and McClane. 2021 Li and McClane. https://creativecommons.org/licenses/by/4.0/ This content is distributed under the terms of the Creative Commons Attribution 4.0 International license.

### Treatment of Caco-2 cells with purified rNanH is sufficient to enhance CPE cytotoxicity and binding.

The results shown in Fig. 4 and 5 indicated that the presence of natural levels of NanH in MDS sporulating culture supernatants increases CPE-induced cytotoxic effects in, and CPE binding to, Caco-2 cells. Since culture supernatants of lysed sporulating cells contain many other factors that might synergistically work with NanH to increase CPE-induced Caco-2 cell cytotoxicity, experiments were also performed to evaluate whether purified recombinant NanH (rNanH) alone is sufficient to affect CPE-induced cytotoxicity.

For this purpose, the *nanH* open reading frame was cloned into the pET45b(+) expression system, and the resultant plasmid was then transformed into Escherichia coli. The same E. coli strain was also transformed with the empty vector for use in a mock purification that served as a negative control. E. coli producing the C. perfringens recombinant CodY (rCodY) protein was used to prepare a second negative control. rNanH and rCodY (Fig. S6A) were highly enriched by metal affinity chromatography purification, and His_6_ tag Western blotting confirmed the identities of the 40-kDa rNanH and the 28-kDa rCodY (Fig. S6A). In contrast, E. coli transformed with pET45b(+) alone (i.e., empty vector) and subjected to mock purification did not produce any proteins that reacted on the His_6_ tag Western blot (Fig. S6A). A sialidase activity assay (Fig. S6B) confirmed that purified rNanH, but not purified CodY or mock-purified material from the empty vector strain, possessed sialidase activity.

10.1128/mSphere.00176-21.6 FIG S6 Recombinant production and purification of rNanH or rCodY or mock purification of similar supernatant volumes from empty vector E. coli. (A) Purified rNanH or rCodY and mock-purified material from an equal volume of empty vector cultures was subjected to SDS-PAGE and then stained with Coomassie blue (left) or Western blotted for 6-His tag anti-mouse (right). (B) Sialidase activity analyses of purified rNanH and rCodY or mock-purified material from empty vector cultures. All experiments were performed in triplicate, and the mean results are shown. Error bars show S.D. *, sialidase activity *P* < 0.05 relative to rNanH. Download FIG S6, TIF file, 0.5 MB. Copyright © 2021 Li and McClane. 2021 Li and McClane. https://creativecommons.org/licenses/by/4.0/ This content is distributed under the terms of the Creative Commons Attribution 4.0 International license.

When these purified samples became available, Caco-2 cells were treated with either 0.5 μg/ml of CPE alone or the same amount of CPE in the presence of three different concentrations of purified rNanH. The sialidase activity of the rNanHM (medium dose of rNanH) sample had equivalent sialidase activity as present in SM101 24-h MDS culture supernatant, the rNanHL (low dose of rNanH) sample had half of the sialidase activity present in that supernatant, and the rNanHH (high dose of rNanH) had twice the sialidase activity of that supernatant. Several controls were also used, including the following: (i) a mock purification using metal affinity chromatography of a culture volume from the empty vector culture that was equivalent to that used for purifying rNanHH, (ii) a molar concentration of rCodY equal to that of rNanHH, or (iii) rNanHH alone.

**FIG 7:**
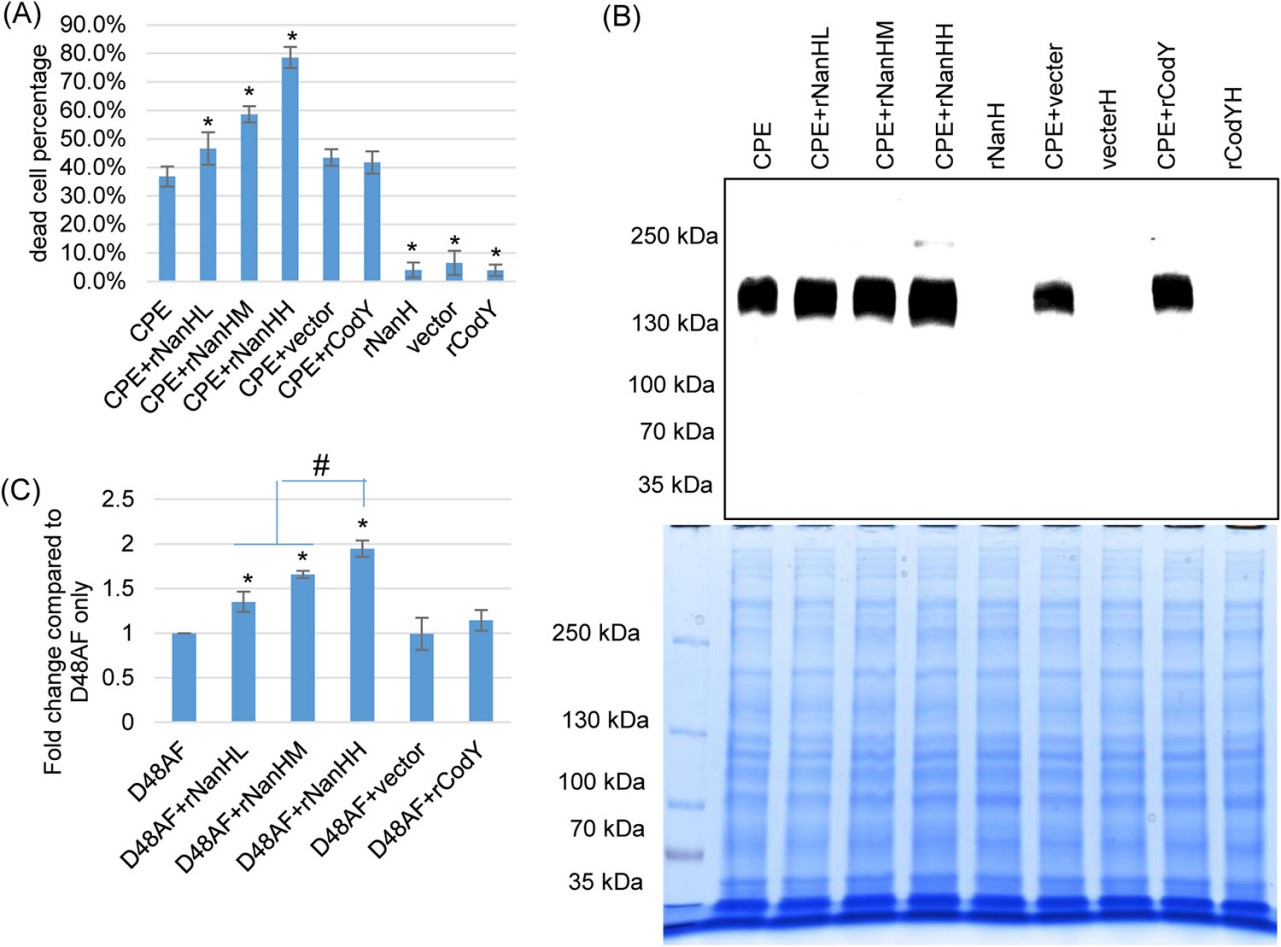
Purified rNanH increases CPE binding, cytotoxicity, and CH-1 large complex formation in Caco-2 cells. (A) Percentage of dead Caco-2 cells after a 1-h treatment at 37°C with 0.5 mg/liter native CPE alone or 0.5 mg/liter CPE in the presence of (i) a low concentration (see Results) of rNanH (rNanHL), (ii) a medium concentration of rNanH (rNanHM), or (iii) a high concentration of rNanH (rNanHH). Also shown is 0.5 mg/liter CPE in the presence of mock-purified material from the same volume of empty vector control E. coli as used to prepare the rNanHH sample from rNanH-producing E. coli or the same molar concentration of purified rCodY as purified rNanHH. Other controls included purified rNanHH, mock-purified empty vector material, and purified rCodY, each in the absence of added CPE. Shown are the mean values from three independent experiments. The error bars indicate the SD. *, *P* < 0.05 relative to CPE alone. (B) CPE large complex in Caco-2 cells. Caco-2 cells were treated with (i) 0.5 mg/liter of native CPE, (ii) 0.5 mg/liter of CPE with rNanHL, rNanHM, or rNanHH, (iii) 0.5 mg/liter of CPE with the mock-purified material from the same volume of empty vector culture as used to obtain a high concentration of rNanH, or (iv) 0.5 mg/liter of CPE plus the molar concentration of purified rCodY equivalent to rNanHH. High-dose rNanH, empty vector, and rCodY alone (no CPE) were served as negative controls. Following incubation, cells were collected and lysed in RIPA buffer, and total proteins were separated by SDS-PAGE and CPE Western blotting was performed (top panel). This blot displays a representative result of three experiments. A duplicate gel was loaded with each sample and stained with Coomassie blue to ensure that samples contained similar amounts of total protein (bottom panel). (C) AF488-CPE_D48A_ (D48AF) binding to Caco-2 cells. Five mg/liter of AF488-CPE_D48A_ was added to purified rNanH (low, medium, or high concentrations), these mixes were then applied to Caco-2 cells for 1 h at 37°C. The cells were then washed three times with HBSS, collected, and lysed in 200 μl RIPA buffer and centrifuged. An aliquot (100 μl) of supernatant was then read by fluorescence at excitation/emission wavelengths 485/519 nm. All data were corrected for background fluorescence by subtraction of fluorescence in similar samples to which AF488-CPE_D48A_ was not added. The value of each bar indicates the calculated fold change in the presence of rNanH, mock-purified empty vector, or rCodY relative to the binding value obtained for AF488-CPE_D48A_ without other additions. Shown are the mean values from three independent experiments; the error bars indicate the SD. *, *P* < 0.05 relative to AF488-CPE_D48A_ in the absence of other purified recombinant proteins; *#*, *P* < 0.05 relative to treatment with the low or medium dose of NanH.

When Caco-2 cells were challenged for 1 h at 37°C, the copresence of purified rNanH and CPE caused significantly more cytotoxicity compared to treatment of these cells with CPE alone (no NanH). Furthermore, this enhancement of CPE cytotoxicity was NanH dose dependent (Fig. 7A). As controls, CPE cytotoxicity was not significantly affected by the copresence of CPE and purified rCodY or the mock-purified sample from the empty vector transformant. Also, treatment of Caco-2 cells with purified rNanH, purified rCodY, or the mock-purified sample from the empty vector transformant alone, i.e., in the absence of CPE, caused little or no increase in cytotoxicity compared to treatment with HBSS buffer alone.

The copresence of purified rNanH and CPE for 1 h at 37°C significantly increased CH-1 complex formation in Caco-2 cells (Fig. 7B). This enhancement was NanH dose dependent (Fig. 7B). As expected, Caco-2 cells treated with purified rNanH, rCodY, or mock-purified empty vector material in the absence of CPE did not form the CH-1 complex, as determined by CPE Western blot analysis.

To test whether the observed enhancement of CH-1 complex in the presence of purified NanH was associated with an increase in CPE binding to Caco-2 cells, Alexa Fluor 488 (AF488)-labeled rCPE_D48A_ was used. When Caco-2 cells were cotreated with purified rNanH and AF488-rCPE_D48A_ at 37°C for 1 h, a significant increase in AF488-rCPE_D48A_ binding was detected (Fig. 7C). This enhancement was rNanH dose dependent (Fig. 7C).

## DISCUSSION

Clostridium perfringens can produce up to three different sialidases, i.e., NanH, NanI, and NanJ (20). C. perfringens type F strains causing FP often do not carry the *nanI* gene encoding NanI, as supported by results of the current and previous studies (23). However, those NanI-negative type F FP strains do consistently carry the *nanH* gene, as also supported by the current and previous results (23). The possible contributions of NanH to the growth, sporulation, and pathogenesis of those type F FP strains have been little studied thus far. Since the absence of a signal peptide on NanH apparently prohibits its active secretion (24, 30), as supported by the current results, why do C. perfringens FP strains produce a cytoplasmic sialidase given that their substrates (sialic acid-containing proteins or lipids) are presumably accessible only to extracellular sialidases?

The current study provides several new insights regarding NanH production and release that suggest a potential pathogenic contribution of NanH sialidase to C. perfringens type F FP. By comparing NanH production in MDS versus TH media, it was determined that NanH is produced predominantly by sporulating cultures of the typical FP strains carrying a chromosomal *cpe* gene. One explanation for this association between NanH production and sporulating cultures was provided by bioinformatic analyses that identified potential Spo0A and SigE binding sites upstream of the *nanH* ORF. The current study then used *spo0A* and *sigE* null mutants to test whether those two proteins are important for NanH production in MDS sporulation medium. Results showed that both Spo0A and SigE are necessary for SM101 spore formation, which is consistent with previous results using SM101 *spo0A* or *sigE* mutants grown using *in vitro* sporulation media (26, 28). In contrast, only Spo0A was determined to be important for strain SM101 to produce NanH in MDS sporulation medium. The *sigE* null mutant still made wild-type levels of NanH, while NanH production by the *spo0A* mutant was significantly less, with complementation reversing this phenotype.

**FIG 8:**
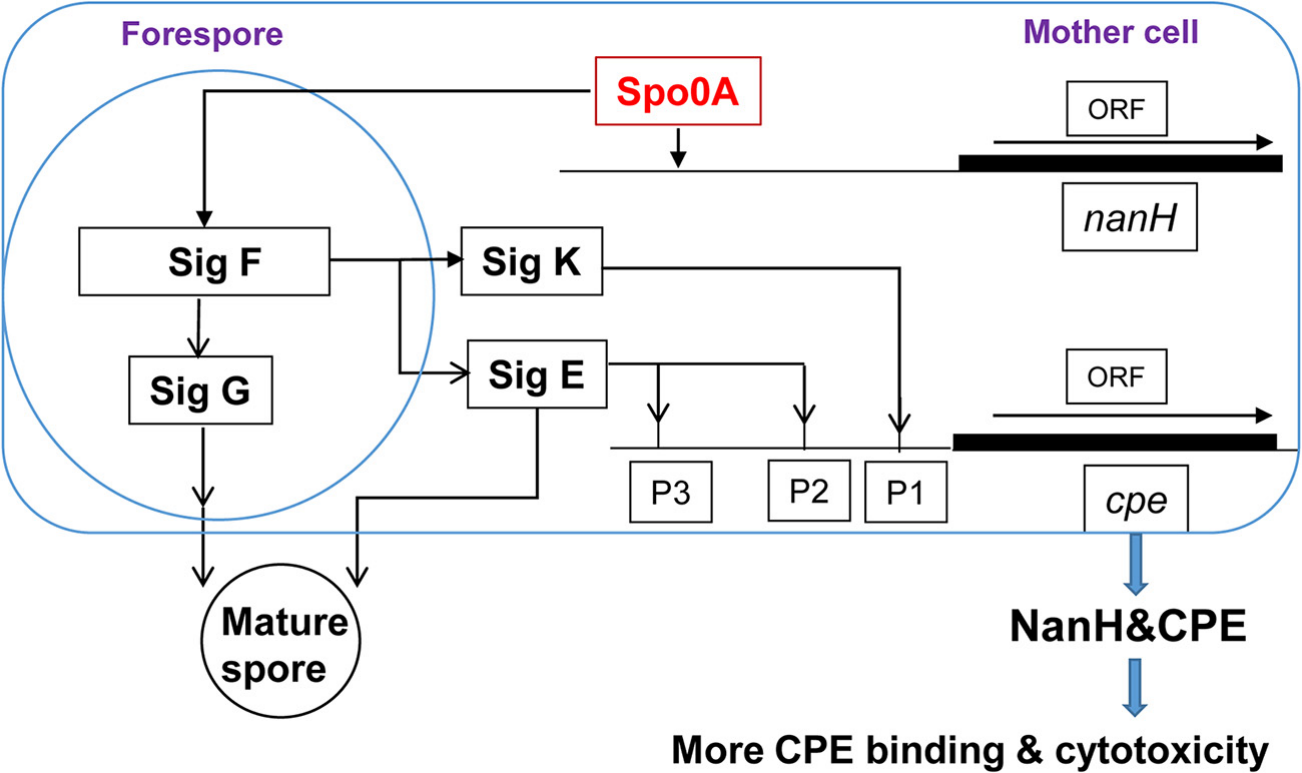
Current model for *nanH* gene regulation and NanH enhancement of CPE action. When C. perfringens enters sporulation, phosphorylated Spo0A upregulates *nanH* gene expression and expression of the four sporulation-associated sigma factors. During sporulation, SigK and SigE upregulate CPE production in the mother cells (28, 43). After maturation of the spore, the mother cell lyses to release its spore, which also results in release of both NanH and CPE. NanH then enhances CPE binding and cytotoxicity. Note that while formation of mature spores requires all four sigma factors (28, 44), that is not true for NanH or CPE production. NanH production does not need SigE (this study), while CPE expression is not dependent on SigG (44). Whether other sporulation-associated sigma factors indirectly regulate NanH production remains to be determined, although there are not readily identified sequences indicating the presence of SigF-, SigG-, or SigK-dependent promoters immediately upstream of the *nanH* gene.

The identification of a role for Spo0A in regulating NanH production when strain SM101 is cultured in MDS medium is informative and leads to the current rudimentary model shown in Fig. 8. However, this regulatory process is likely to be complex and may differ under different experimental conditions. In addition, it may involve other regulatory proteins. For example, bioinformatics identified binding motifs for other regulatory proteins upstream of *nanH*. These included binding sites for CodY and NanR, which have also been linked to the regulation of sporulation in C. perfringens (31, 32). Future studies should investigate these and other regulators in controlling NanH production by type F FP strains.

Coupled with previous studies (33, 34), the current results also revealed some apparent differences between the regulation of NanH production by type F FP strains versus other C. perfringens strains. Previous studies with C. perfringens type D strain CN3718 showed that a *nanI nanJ* double mutant of this strain produces significant amounts of NanH by 8 h of culture in TH broth, although that NanH was almost completely cytoplasmic at that time point (33, 34). Interestingly, more NanH sialidase activity was detectable in 24-h supernatants of TH versus MDS cultures of this CN3718 *nanI* and *nanJ* double mutant, despite a near total lack of sporulation (<10 spores/ml) in the 24-h TH culture of this strain. These differences between the regulation of NanH production by type F FP strains like SM101 versus type D strain CN3718 extend previous reports of regulatory differences between these strains (31), e.g., CodY represses sporulation by CN3718 but increases SM101 sporulation. These regulatory differences in NanH regulation and the role of CodY in regulating sporulation between strains SM101 and CN3718, plus the absence of *nanI* and *pfoA* from type F FP strains like SM101, add further support to the emerging view of type F FP strains with a chromosomal *cpe* gene (and certain type C strains) as representing a distinct sublineage of C. perfringens (18, 19).

The observed linkage between NanH production and sporulating cultures of strain SM101 might have suggested that NanH can provide type F FP strains with nutrients needed for growth under sporulating conditions or for completion of sporulation. However, studies using a *nanH* null mutant showed that NanH is not necessary for the growth or sporulation of type F FP strain SM101, at least in MDS sporulation medium. These results are consistent with the determination in this study that MDS cultures of SM101 do not release NanH extracellularly (to encounter sialylated proteins or lipids) until the completion of sporulation, by which time it would not be helpful for contributing to either growth or sporulation.

The current study does suggest one explanation for how type F FP strains (and perhaps type F NFD strains) could benefit from NanH production. Specifically, in sporulating cultures, the extracellular release of NanH and CPE was shown to coincide. That observation is significant because extracellular CPE release is known to occur only when mother cells lyse to release their mature spore (10). Therefore, mother cell lysis provides an extracellular release mechanism for both NanH and CPE, two proteins lacking a secretion signal peptide. This extracellular release of NanH via lysis of the mother cell could benefit FP strains by impacting their pathogenicity. The simultaneous extracellular presence of both CPE and NanH could increase CPE activity during FP, as supported by *in vitro* results presented in this study. Specifically, the presence of NanH in lysed SM101 sporulating culture lysates caused a nearly twofold enhancement in CPE-induced cytotoxicity for Caco-2 cells. This effect does not require the presence of other factors in sporulating culture lysates since purified rNanH alone was sufficient to cause a similar enhancement of CPE activity. The mechanism behind rNanH enhancement of CPE cytotoxicity was shown to involve promotion of CPE binding, which allowed more formation of the CH-1 pore. This NanH-mediated increase in CPE binding could result from NanH reducing host cell surface charge repulsion during CPE binding or from NanH removing sialic acid residues that sterically interfere with CPE binding.

While a twofold enhancement of CPE activity by NanH may appear modest, it nonetheless could have pathogenic impact. Many type F FP strains produce less than the ∼50 μg/ml of CPE needed in a purified form to cause pathological effects in intestinal loops of animal models (35, 36). A twofold enhancement of CPE activity *in vivo* by NanH could sufficiently increase CPE activity to confer enteric virulence to many type F FP strains. Future studies will test this hypothesis *in vivo*.

It was previously shown that, when produced, NanI sialidase can increase the binding and cytotoxicity of three C. perfringens toxins, i.e., CPE and epsilon toxin and beta toxin (21, 33). The current findings now expand upon that previous work by showing that other C. perfringens sialidases can also impact the binding and activity of toxins that play major roles in C. perfringens disease. However, as mentioned in the introduction, NanI has also been shown to be an important contributor to other potentially important steps in pathogenesis. For example, secretion of NanI promotes the persistent intestinal colonization of mice by vegetative cells of type F NFD strains, which likely explains why type F NFDs can last up to several weeks (22). The absence of NanI production by many type F FP strains like SM101 would likely impair intestinal colonization by their vegetative cells and may explain why type F FP is of much shorter duration than type F NFD. In this hypothesis, the inability of type F FP strains like SM101 to extracellularly release their only sialidase (NanH) until the completion of sporulation could be a further explanation for the short duration of type F FP.

Last, it is interesting that NanI and NanH both enhance CPE binding even though NanH activity shows a preference for α-2,8 > α-2,3 > α-2,6 sialic acid linkages, while NanI shows preferential activity for α-2,3 > α-2,6 > α-2,8 sialic acid linkages (34). Given those differences in substrate preferences, and the possible coproduction of NanI and NanH by type F NFD and some FP strains, future studies should investigate whether NanI and NanH together can act synergistically to further promote CPE binding and activity *in vivo*. Also, the role of NanJ (when produced) in C. perfringens growth, sporulation, and pathogenicity remains to be explored.

## MATERIALS AND METHODS

### Bacterial media and chemicals.

Media used in this study for preparing C. perfringens stock cultures included cooked meat medium (CMM; Difco Laboratories) and FTG medium (fluid thioglycolate medium; Difco Laboratories). To obtain sporulating cultures, C. perfringens strains were grown in MDS sporulation medium (proteose peptone [15 g/liter], yeast extract [4 g/liter], sodium thioglycolate [1 g/liter], disodium phosphate [10 g/liter], raffinose [4 g/liter], and caffeine [19.2 g/liter]) (37). To grow predominantly vegetative cultures of C. perfringens, TH medium (Bacto Todd-Hewitt broth [Becton-Dickinson] with 0.1% sodium thioglycolate [Sigma-Aldrich]) and TGY medium (3% tryptic soy broth [Becton-Dickinson] with 2% glucose [Fisher Scientific], 1% yeast extract [Becton-Dickinson], and 0.1% sodium thioglycolate [Sigma-Aldrich]) were used. For mutant selection and spore counting, BHI (brain heart infusion; Becton-Dickinson) agar plates with or without chloramphenicol (Cm) (Sigma-Aldrich) were utilized. Media employed for culturing E. coli included Luria-Bertani (LB) broth (1% tryptone [Becton-Dickinson], 0.5% yeast extract [Becton-Dickinson], 1% NaCl [Fisher Scientific]) and LB agar (1.5% agar [Becton-Dickinson]). Ampicillin (Am) was purchased from Fisher Scientific Company. Isopropyl-β-d-thiogalactopyranoside (IPTG) was purchased from CHEM-IMPEX INT’L Inc.

### Bacterial strains.

C. perfringens strains used in this study are listed in Table 1 and included nine wild-type, chromosomal *cpe*, type F FP strains, as well as a previously constructed (26) *spo0A* null mutant, named IH101, of type F FP strain SM101 and its complementing strain named IH101(MRS123). DNA from C. perfringens CN3718, a type D animal disease strain that produces epsilon toxin (ETX) and all three sialidases (NanJ, NanI, and NanH) (33) was utilized as a positive control for the sialidase PCR and Southern blot analyses. Shuttle plasmids pJIR750ai and pJIR750 (27, 38) were used for constructing *nanH*, *cpe*, and *sigE* knockout mutants or *nanH* and *sigE* complementing strains, respectively.

**TABLE 1:** C. perfringens strains used in this study

Isolate	Description (disease, location/date of isolation)	Reference
NCTC8239	Food poisoning, Europe 1950s	Collie et al. (35)
NCTC10239	Food poisoning, Europe 1950s	Collie et al. (35)
SM101	Food poisoning, Europe 1950s	Zhao and Melville (43)
C1841	Food poisoning, Vermont 1980s	Sparks et al. (17)
FD1041	Food poisoning, North America, 1980s	Sparks et al. (17)
E13	Food poisoning, North America, 1960s	Sparks et al. (17)
527	Food poisoning, North America, 1990s	Sparks et al. (17)
01E803	Food poisoning, Oklahoma, 1999	Bos et al. (7)
01E809	Food poisoning, Oklahoma, 1999	Bos et al. (7)
IH101	SM101 *spo0A* null mutant strain	Huang et al (26)
IH101(MRS123)	SM101 *spo0A* complemented strain	Huang et al (26)
SM101*nanH*KO	SM101 *nanH* null mutant strain	This study
SM101*nanHc*	SM101 *nanH* complemented strain	This study
SM10*cpe*KO	SM101 *cpe* null mutant strain	This study
SM101DKO	SM101 *nanH* and *cpe* double null mutant strain	This study
DKO*nanH*c	SM101 DKO *nanH* complemented strain	This study
SM101*sigE*KO	SM101 *sigE* null mutant strain	This study
SM101*sigEc*	SM101 *sigE* complemented strain	This study

In this study, all C. perfringens strains were cultured at 37°C under anaerobic conditions.

### Recombinant protein purification.

The open reading frame (ORF) encoding recombinant NanH (rNanH) was synthesized by GenScript and cloned into expression plasmid pET-45b(+) between the KpnI and AvrII sites. Empty vector pET-45b(+)-Novagen was purchased from Sigma-Aldrich. After those two plasmids were separately transformed into E. coli BL21(DE3) (purchased from New England Biolabs [NEB]), rNanH protein was purified as previously described for rNanI purification (21).

To purify rCPE_D48A,_ which is a noncytotoxic recombinant CPE variant that binds to cells but does not oligomerize or form pores (29), the rCPE_D48A_ ORF was synthesized by GenScript and cloned into plasmid pET-45b(+) between the SacI and AvrII sites. After the resultant plasmid was transformed into E. coli HMS147 (Novagen), the rCPE_D48A_ protein was purified as described previously (29).

Preparation of a plasmid encoding recombinant CodY protein (rCodY) and protein purification of that recombinant C. perfringens protein were described previously (39).

Native CPE was purified to homogeneity from C. perfringens strain NCTC 8238 (ATCC 12916), as described previously for strain NCTC 8239 (40). All proteins were confirmed by His_6_ tag Western blotting or CPE Western blotting, as appropriate (see below).

### Construction of SM101 null mutants and complementing strains.

The *nanH*, *cpe*, or *sigE* gene in type F FP strain SM101 was inactivated by insertion of a targeted group II intron using the *Clostridium*-modified Targetron system (27). A 350-bp intron-targeting product was constructed by PCR using the pACD4k-C plasmid (Sigma-Aldrich) as the template and the primers nanH-422|423a-IBS, nanH-422|423a-EBS1d, and nanH-422|423a-EBS2 (Table 2). This product was then inserted into pJIR750ai between the HindIII and BsrGI enzyme sites to construct the pJIR750(SM101)*nanH*i vector. This plasmid inactivates the *nanH* gene by causing insertion of a targeted group II intron between nucleotides 422 and 423 of the *nanH* ORF. The previously prepared intron-targeting plasmid pJIR750*cpe*i (41), which inactivates the *cpe* gene by inserting an intron between nucleotides 195 and 196 of the *cpe* ORF, was used to construct a *cpe* null mutant of strain SM101. For constructing a *sigE* gene null mutant of SM101, a 350-bp intron-targeting product was constructed by PCR using the pACD4k-C plasmid as the template and the primers sigE-171|172a-IBS, sigE-171|172a-EBS1d, and sigE-171|172a-EBS2 (Table 2). The resultant product was then inserted into pJIR750ai between the HindIII and BsrGI enzyme sites to construct the pJIR750*sigE*i vector. This plasmid inactivates the *sigE* gene by causing the insertion of an intron between nucleotides 171 and 172 of the *sigE* ORF.

**TABLE 2:** Primers used in this study

Primer name	Primer sequence	Purpose	PCR product size (bp)
nanH-422|423a -IBS	AAAAAAGCTTATAATTATCCTTACTTCTCCTTGCAGTGCGCCCAGATAGGGTG	pJIR750*nanH*i construction	350
nanH-422|423a -EBS1d	CAGATTGTACAAATGTGGTGATAACAGATAAGTCCTTGCAGATAACTTACCTTTCTTTGT	pJIR750*nanH*i construction	350
nanH-422|423a -EBS2	TGAACGCAAGTTTCTAATTTCGGTTAGAAGTCGATAGAGGAAAGTGTCT	pJIR750*nanH*i construction	350
EBS universal	CGAAATTAGAAACTTGC GTTCAGTAAAC	pJIR750*nanH*i construction	350
cpeKOF	GGAGATGGTTGGATATTAGG	Screen for intron insertion in *cpe*	233
cpeKOR	GGACCAGCAGTTGTAGATA	Screen for intron insertion in *cpe*	233
nanHFnew	TCTGCACGCAGTACTGATTTT	Screen for intron insertion in *nanH*	314
nanHRnew	CCTAGCCATCCAATTGTATTACTTG	Screen for intron insertion in *nanH*	314
qnanHF	TGCAGGCTCATGGAATACAA	qRT-PCR for *nanH*	72
qnanHR	TGGACAGACCAATCACTTCTTC	qRT-PCR for *nanH*	72
nanHcomF	CGGCGGATCCTTTATAATAATTCTAAGTCTCACC	*nanH* complementation	2,115
nanHcomR	GCAGGTCGACGAAAAGAGTCTGTCATTAGCAG	*nanH* complementation	2,115
sigE-171|172-IBS	AAAAAAGCTTATAATTATCCTTAATTAGCAGTATTGTGCGCCCAGATAGGGTG	pJIR750*sigE*i construction	350
sigE-171|172-EBS1d	CAGATTGTACAAATGTGGTGATAACAGATAAGTCAGTATTCTTAACTTACCTTTCTTTGT	pJIR750*sigE*i construction	350
sigE-171|172-EBS2	TGAACGCAAGTTTCTAATTTCGGTTCTAATCCGATAGAGGAAAGTGTCT	pJIR750*sigE*i construction	350
SigEKOF	TTACGAATTCTTTACAAGAGAGTAATATTATCAGC	Screen for intron insertion in *sigE* RT-PCR	236
SigEKOR	AACTCCTACACCAGTGTTCTC	Screen for intron insertion in *sigE* RT-PCR	236
sigEcomF	TTACGAATTCTTTACAAGAGAGTAATATTATCAGC	*sigE* complementation	1,453
sigEcomR	TAGAGGATCCAGAGGTTAATTTTATACTCTTAAATC	*sigE* complementation	1,453

To construct *nanH*, *cpe*, or *sigE* single mutant strains, plasmids pJIR750(SM101)*nanH*i, pJIR750*cpe*i, or pJIR750*sigE*i were electroporated into wild-type SM101. To construct a *cpe* and *nanH* double null mutant, plasmid pJIR750*cpe*i was electroporated into the *nanH* null mutant strain. All mutants were selected using BHI agar plates containing 15 mg/liter Cm. The *nanH* null mutant primers used for screening were nanH(A)F and nanH(A)R; for screening *cpe* null mutants, the primers used were cpeKOF and cpeKOR, and for screening a *sigE* null mutant, the primers employed were sigEKOF and sigEKOR (Table 2). Transformants were PCR screened for an intron-disrupted *nanH*, *cpe* or *sigE* gene as described in Results. The obtained *nanH* null mutant was named SM101*nanH*KO, the *cpe* null mutant was named SM101*cpe*KO, the *nanH* and *cpe* double null mutant was named SM101DKO, and the *sigE* null mutant was named SM101*sigE*KO.

For this study, to create a *nanH* complementing strain named SM101nanHcomp, two primers named nanHcomF and nanHcomR (Table 2) were used to amplify a 2,115-bp *nanH* PCR product that contains 454 bp of sequence upstream of the *nanH* start codon. To create a *sigE* complementing strain named SM101sigEcomp, two primers named sigEcomF and sigEcomR (Table 2) were used to amplify a 1,453-bp *sigE* PCR product that contains about 500 bp of sequence upstream of the *sigE* start codon. SM101 genomic DNA was served as the template DNA. The PCR amplification conditions used for these amplifications were: 1 cycle of 95°C for 2 min; 35 cycles with 1 cycle consisting of 95°C for 30 s, 55°C for 40 s, and 72°C for 2 min; and a single extension of 72°C for 5 min. Each *nanH* complementing PCR product was cloned into the C. perfringens*/*E. coli shuttle plasmid pJIR750 between the BamHI and SalI sites. Each *sigE* complementing PCR product was cloned into pJIR750 between the EcoRI and BamHI sites. After electroporation of the resultant plasmids, complementing strains was selected by BHI agar plates containing 15 mg/liter of Cm.

### PCR analyses of sialidase gene carriage.

PCR was performed to survey the presence of the following genes in type F FP strains: (i) the *nanJ* gene, using the nanJKOF and nanJKOR primers (33); (ii) the *nanI* gene, using primers nanIKOF and nanIKOR (33); and (iii) the *nanH* gene, using primers nanHKOF and nanHKOR (33). These PCR mixtures included 1 μl of each pair of primers (at a 0.5 μM final concentration), 1 μl of purified DNA template (100 ng), and 25 μl of 2× DreamTaq Green PCR Master Mix (Fisher Scientific), which were mixed together before double-distilled H_2_O (ddH_2_O) was added to reach a total volume of 50 μl. The reaction mixtures were placed in a thermal cycler (Techne) and subjected to the following amplification conditions: 1 cycle of 95°C for 2 min; 35 cycles with 1 cycle consisting of 95°C for 30 s, 55°C for 40 s, and 72°C for 40 s; and a single extension of 72°C for 5 min. PCR products were then electrophoresed on a 1.5% agarose gel, which was stained with ethidium bromide.

### Southern blot analyses of sialidase gene carriage.

A *nanI*-specific probe was prepared by PCR using the primers NanIprobF and NanIprobR (33). Similarly, a *nanJ*-specific probe was prepared by PCR using the primers NanJprobF and NanJprobR (33), and a *nanH*-specific probe was prepared by PCR using the primers nanHKOF and nanHKOR (33). CN3718 DNA was used as the template DNA for all three PCRs. Last, for preparing an intron-specific probe, PCR was carried out using the primers from nanH-422|423a-IBS and nanH-422|423a-EBS1d, with pJIR750nanH(A)i plasmid DNA serving as the template. These PCR mixtures included 1 μl of each pair of primers (at a 0.5 μM final concentration), 1 μl of purified DNA template (100 ng), and 25 μl of 2×*Taq* Mixture (NEB), which were mixed together before ddH_2_O was added to reach a total volume of 50 μl. The reaction mixtures were then placed in a thermal cycler (Techne) and subjected to the following amplification conditions: 1 cycle of 95°C for 2 min; 35 cycles with 1 cycle consisting of 95°C for 30 s, 55°C for 40 s, and 68°C for 40 s; and a single extension of 68°C for 5 min. PCR products were then gel purified and labeled using the PCR DIG Labeling kit (Roche Applied Science) according to the manufacturer’s instructions.

Using the Epicentre DNA purification kit, DNA was isolated from the surveyed C. perfringens wild-type FP strains or SM101 null mutant strains. For a positive control, DNA was purified from the *cpe-*negative, type D strain CN3718, a strain that carries all three sialidase genes (33). An aliquot of each purified DNA (3 μg) was digested overnight at 37°C with BsrGI or EcoRI, as indicated in Results, and then electrophoresed on a 1% agarose gel. After alkali transfer to a nylon membrane (Roche), the blot was hybridized with a digoxigenin-labeled, *nanJ*-specific probe as described previously (33). After stripping off the *nanJ* probe (33), the blot was rehybridized with a *nanI-* or *nanH*-specific probe. Digoxigenin (DIG) detection reagents were purchased from Roche Applied Science. Disodium 3-(4-methoxyspiro {1,2-dioxetane-3,2'-(5'-chloro)tricyclo [3.3.1.13,7]decan}-4-yl)phenyl phosphate (CSPD) substrate (Roche) was used for detection of hybridized probes according to the manufacturer’s instructions.

For intron Southern blot analysis of mutant strains, an intron-specific probe was used.

### C. perfringens mRNA isolation and qRT-PCR analysis.

Strain SM101 was grown in TH or MDS broth for 3 h at 37°C. RNA was then extracted from pelleted cultures using saturated phenol and purified by TRIzol and chloroform (Life Technologies and Sigma) as previously described (42). The isolated RNA was confirmed by PCR without reverse transcriptase (RT) to be DNA free before quantitative reverse transcriptase PCR (qRT-PCR) analysis. If any DNA contamination was detected, DNase (Thermo Fisher) was used to remove the residual DNA contamination. The purified RNA was then quantified by determining the absorbance at 260 nm, and cDNA was prepared using a Maxima first-strand cDNA synthesis kit (Thermo Scientific) according to the manufacturer’s instructions. The *nanH* qRT-PCR primers were designed using the Integrated DNA Technologies (IDT) website and are listed in Table 2. Each cDNA was diluted 10 times to 5 ng/μl. Power SYBR green PCR master mix (Thermo Fisher Scientific) and a StepOnePlus qRT-PCR instrument (Applied Biosystems) were used to perform qRT-PCR as described in a previous paper (42). After qRT-PCR, the relative quantitation of mRNA expression was normalized to the level of constitutive expression of the housekeeping 16S RNA and calculated by the comparative threshold cycle (2^−ΔΔCT^) method (42).

### C. perfringens growth curve and quantitative counts of heat-resistant spores.

Measurement of C. perfringens growth (optical density at 600 nm [OD_600_]), and quantitation of heat-resistant spore formation was performed as described previously (42).

### Sample preparation for sialidase enzyme activity and CPE Western blot analysis.

TH or MDS cultures were or were not sonicated with a Qsonica sonicator. The sonication program was six cycles of 10-s sonication, followed by 30-s rest with sonicator output set at 30%. The sonicated sample volume was 4 ml, and sonication was performed on ice. All samples were centrifuged, and the supernatants were used for sialidase activity or CPE Western blotting.

### Measurement of sialidase enzyme activity.

A 0.2-ml aliquot of a FTG overnight culture was transferred to 10 ml of fresh TH or MDS medium, and those cultures were then incubated at 37°C for different time points as indicated (see Results). A 60-μl aliquot of supernatant from each culture was added to a 40-μl aliquot of substrate (4 mM 5-bromo-4-chloro-3-indolyl-α-d-*N*-acetylneuraminic acid [Santa Cruz]), and the mixture was incubated at 37°C for 1 h. The absorbance at 595 nm was then measured using a Bio-Rad microplate reader.

### Western blot analyses.

To evaluate CPE production, a 0.2-ml aliquot sample from an overnight FTG culture of a C. perfringens wild-type, null mutant, or complementing strain was inoculated into 10 ml of MDS or TH medium for indicated times. To perform a CPE Western blot assay, culture aliquots were removed, and supernatants or sonicated whole culture were assayed as specified. After mixing with 5× sodium dodecyl sulfate (SDS) loading buffer, a CPE anti-rabbit polyclonal antibody was used to perform CPE Western blotting as described previously (23). The same samples were loaded on another SDS-acrylamide gel and stained with Coomassie blue G250 to assess equivalent sample protein content.

To perform a recombinant His_6_-tagged protein Western blot assay, an aliquot of purified protein was mixed with 5× SDS loading buffer, and a His_6_ tag anti-mouse monoclonal antibody (R&D Systems) was used to perform the blotting (21). The same samples were loading to another SDS gel and stained with Coomassie blue G250 to ensure that the recombinant proteins were not contaminated with other E. coli proteins.

### Evaluation of CPE-induced cytotoxicity and CPE large complex formation.

Caco-2 cells were cultured as described previously (40). Confluent Caco-2 cells grown in 12-well plates were treated for 1 h with Hanks balanced salt solution with calcium and magnesium without phenol red (HBSS; Corning) containing buffer-exchanged 1-ml MDS culture supernatants of wild-type, its *nanH* null mutant, a *nanH* complementing strain, a *cpe* null mutant, or its *nanH cpe* double null mutant. MDS culture supernatants were buffer exchanged into HBSS using Thermo Scientific Zeba spin desalting columns. Confluent Caco-2 cells grown in 12-well plates were also treated for 1 h with HBSS containing 0.5 μg/ml native CPE in the presence or absence of purified rNanH, rCodY, or vector proteins, as specified. HBSS served as a negative control. Following this treatment, the supernatant was removed for cytotoxicity detection using the Roche cytotoxicity detection kit (LDH). Caco-2 cells were then gently removed from plates and resuspended in radioimmunoprecipitation assay (RIPA) buffer (Alfa Aesar) containing Benzonase (Millipore Sigma) and proteinase inhibitor (Research Products International [RPI]). Samples in 5× SDS loading buffer were loaded onto 6% SDS-acrylamide gels for CPE Western blotting or to show equal protein content in samples. Three doses of purified rNanH were used, i.e., a low (rNanHL), medium (rNanHM), and high (rNanHH) concentration. rNanHH is equivalent to twice the sialidase activity in SM101 MDS 24-h culture supernatant, rNanHM is equivalent to the sialidase activity in SM101 MDS 24-h culture supernatant, and rNanHL is equivalent to half the sialidase activity in SM101 MDS 24-h culture supernatant. rCodY was used at the same molar concentration as rNanHH.

### Alexa Fluor 488 (AF488) labeling of rCPE_D48A_ and use of AF488-labeled rCPE_D48_A in a Caco cell binding assay.

To determine whether NanH can affect the ability of CPE to bind to Caco-2 cells, confluent monolayers of Caco-2 cell were incubated at 37°C for 1 h with 5 μg/ml of AF488-labeled rCPE_D48A_ in 1 ml of buffer-exchanged 24-h MDS culture supernatants of the *cpe* null mutant, *cpe nanH* double mutant, or *nanH* complemented double knockout (DKO) strain. In other experiments, Caco-2 cells were treated for 1 h at 37°C with 5 μg/ml of AF488-labeled rCPE_D48A_ in 1 ml of HBSS buffer containing rNanH or rCodY (at the same molar concentration as rNanHH) or proteins purified from an equal volume of the empty vector strain as a control. After a 1-h treatment, the cells were washed three times with HBSS and then lysed in 200 μl RIPA buffer. A 100-μl aliquot of supernatant was transferred to a 96-well plate, and fluorescence was read at 428/529 nm using a BioTek Synergy plate reader. The background fluorescence in the no AF488-labeled rCPE_D48A_ samples was subtracted from that of the matching samples treated with labeled rCPE_D48A_.

### Statistical analyses.

All statistical analyses were performed using GraphPad Prism 8. For comparison of more than two samples, one-way analysis of variance (ANOVA) was applied with *post hoc* analysis by Dunnett’s multiple-comparison test. For comparison of two samples, Student’s *t* test was applied. Differences were considered significant when the *P* value was less than 0.05.

## References

[1] McClane BA, Robertson SL, Li J. 2013. *Clostridium perfringens*, p 465−489. In Doyle MP, Buchanan RL (ed), Food microbiology: fundamentals and frontiers, 4th ed. ASM Press, Washington, DC.

[2] CDC. 2011. CDC estimates of foodborne illness in the United States: *Clostridium perfringens*. Centers for Disease Control and Prevention, Atlanta, GA. https://www.cdc.gov/foodborneburden/pdfs/FACTSHEET_A_FINDINGS.pdf.

[3] Scallan E, Hoekstra RM, Angulo FJ, Tauxe RV, Widdowson MA, Roy SL, Jones JL, Griffin PM. 2011. Foodborne illness acquired in the United States–major pathogens. Emerg Infect Dis 17:7–15. doi:10.3201/eid1701.p11101. 21192848 PMC3375761

[4] Bamford C, Milligan P, Kaliski S. 2019. Dangers of *Clostridium perfringens* food poisoning in psychiatric patients. S Afr J Psychiatr 25:1339. doi:10.4102/sajpsychiatry.v25i0.1339. 32201630 PMC7081833

[5] Sarker MR, Carman RJ, McClane BA. 1999. Inactivation of the gene (*cpe*) encoding *Clostridium perfringens* enterotoxin eliminates the ability of two *cpe*-positive *C. perfringens* type A human gastrointestinal disease isolates to affect rabbit ileal loops. Mol Microbiol 33:946–958. doi:10.1046/j.1365-2958.1999.01534.x. 10476029

[6] Navarro MA, McClane BA, Uzal FA. 2018. Mechanisms of action and cell death associated with *Clostridium perfringens* toxins. Toxins (Basel) 10:212. doi:10.3390/toxins10050212. PMC5983268 29786671

[7] Bos J, Smithee L, McClane B, Distefano RF, Uzal F, Songer JG, Mallonee S, Crutcher JM. 2005. Fatal necrotizing colitis following a foodborne outbreak of enterotoxigenic *Clostridium perfringens* type A infection. Clin Infect Dis 40:e78–e83. doi:10.1086/429829. 15844055

[8] Centers for Disease Control and Prevention. 2012. Fatal foodborne *Clostridium perfringens* illness at a state psychiatric hospital–Louisiana. MMWR Morb Mortal Wkly Rep 61:605–608. 22895383

[9] Li J, Paredes-Sabja D, Sarker MR, McClane BA. 2016. *Clostridium perfringens* sporulation and sporulation-associated toxin production. Microbiol Spectr 4(3):10.1128/microbiolspec.TBS-0022-2015. doi:10.1128/microbiolspec.TBS-0022-2015. PMC4920134 27337447

[10] Duncan CL. 1973. Time of enterotoxin formation and release during sporulation of *Clostridium perfringens* type A. J Bacteriol 113:932–936. doi:10.1128/JB.113.2.932-936.1973. 4347930 PMC285311

[11] Carman RJ. 1997. *Clostridium perfringens* in spontaneous and antibiotic-associated diarrhoea of man and other animals. Rev Med Microbiol 8:S43–S45.

[12] McDonel JL. 1986. Toxins of *Clostridium perfringens* types A, B, C, D, and E, p 477−517. *In* Dorner F, Drews H (ed), Pharmacology of bacterial toxins. Pergamon Press, Oxford, United Kingdom.

[13] Uzal FA, Freedman JC, Shrestha A, Theoret JR, Garcia J, Awad MM, Adams V, Moore RJ, Rood JI, McClane BA. 2014. Towards an understanding of the role of *Clostridium perfringens* toxins in human and animal disease. Future Microbiol 9:361–377. doi:10.2217/fmb.13.168. 24762309 PMC4155746

[14] Rood JI, Adams V, Lacey J, Lyras D, McClane BA, Melville SB, Moore RJ, Popoff MR, Sarker MR, Songer JG, Uzal FA, Van Immerseel F. 2018. Expansion of the *Clostridium perfringens* toxin-based typing scheme. Anaerobe 53:5–10. doi:10.1016/j.anaerobe.2018.04.011. 29866424 PMC6195859

[15] Cornillot E, Saint-Joanis B, Daube G, Katayama S, Granum PE, Canard B, Cole ST. 1995. The enterotoxin gene (*cpe*) of *Clostridium perfringens* can be chromosomal or plasmid-borne. Mol Microbiol 15:639–647. doi:10.1111/j.1365-2958.1995.tb02373.x. 7783636

[16] Collie RE, McClane BA. 1998. Evidence that the enterotoxin gene can be episomal in *Clostridium perfringens* isolates associated with nonfoodborne human gastrointestinal diseases. J Clin Microbiol 36:30–36. doi:10.1128/JCM.36.1.30-36.1998. 9431915 PMC124802

[17] Sparks SG, Carman RJ, Sarker MR, McClane BA. 2001. Genotyping of enterotoxigenic *Clostridium perfringens* isolates associated with gastrointestinal disease in North America. J Clin Microbiol 39:883–888. doi:10.1128/JCM.39.3.883-888.2001. 11230399 PMC87845

[18] Deguchi A, Miyamoto K, Kuwahara T, Miki Y, Kaneko I, Li J, McClane BA, Akimoto S. 2009. Genetic characterization of type A enterotoxigenic *Clostridium perfringens* strains. PLoS One 4:e5598. doi:10.1371/journal.pone.0005598. 19479065 PMC2682570

[19] Ma M, Li J, McClane B. 2012. Genotypic and phenotypic characterization of *Clostridium perfringens* isolates from Darmbrand cases in post-World War II Germany. Infect Immun 80:4354−4363. doi:10.1128/IAI.00818-12. 23027533 PMC3497428

[20] Li J, Uzal FA, McClane BA. 2016. *Clostridium perfringens* sialidases: potential contributors to intestinal pathogenesis and therapeutic targets. Toxins (Basel) 8:341. doi:10.3390/toxins8110341. PMC5127137 27869757

[21] Theoret JR, Li J, Navarro MA, Garcia JP, Uzal FA, McClane BA. 2017. Native or proteolytically activated NanI sialidase enhances the binding and cytotoxic activity of *Clostridium perfringens* enterotoxin and beta toxin. Infect Immun 86:e00730-17. doi:10.1128/IAI.00730-17. 29038129 PMC5736825

[22] Navarro MA, Li J, McClane BA, Morrell E, Beingesser J, Uzal FA. 2018. NanI sialidase is an important contributor to *Clostridium perfringens* type F strain F4969 intestinal colonization in mice. Infect Immun 86:e00462-18. doi:10.1128/IAI.00462-18. 30297524 PMC6246908

[23] Li J, McClane BA. 2014. Contributions of NanI sialidase to Caco-2 cell adherence by *Clostridium perfringens* type A and C strains causing human intestinal disease. Infect Immun 82:4620–4630. doi:10.1128/IAI.02322-14. 25135687 PMC4249343

[24] Myers GS, Rasko DA, Cheung JK, Ravel J, Seshadri R, DeBoy RT, Ren Q, Varga J, Awad MM, Brinkac LM, Daugherty SC, Haft DH, Dodson RJ, Madupu R, Nelson WC, Rosovitz MJ, Sullivan SA, Khouri H, Dimitrov GI, Watkins KL, Mulligan S, Benton J, Radune D, Fisher DJ, Atkins HS, Hiscox T, Jost BH, Billington SJ, Songer JG, McClane BA, Titball RW, Rood JI, Melville SB, Paulsen IT. 2006. Skewed genomic variability in strains of the toxigenic bacterial pathogen, *Clostridium perfringens*. Genome Res 16:1031–1040. doi:10.1101/gr.5238106. 16825665 PMC1524862

[25] Czeczulin JR, Hanna PC, McClane BA. 1993. Cloning, nucleotide sequencing, and expression of the *Clostridium perfringens* enterotoxin gene in *Escherichia coli*. Infect Immun 61:3429–3439. doi:10.1128/IAI.61.8.3429-3439.1993. 8335373 PMC281020

[26] Huang IH, Waters M, Grau RR, Sarker MR. 2004. Disruption of the gene (*spo0A*) encoding sporulation transcription factor blocks endospore formation and enterotoxin production in enterotoxigenic *Clostridium perfringens* type A. FEMS Microbiol Lett 233:233–240. doi:10.1016/j.femsle.2004.02.014. 15063491

[27] Chen Y, McClane BA, Fisher DJ, Rood JI, Gupta P. 2005. Construction of an alpha toxin gene knockout mutant of *Clostridium perfringens* type A by use of a mobile group II intron. Appl Environ Microbiol 71:7542–7547. doi:10.1128/AEM.71.11.7542-7547.2005. 16269799 PMC1287605

[28] Harry KH, Zhou R, Kroos L, Melville SB. 2009. Sporulation and enterotoxin (CPE) synthesis are controlled by the sporulation-specific sigma factors SigE and SigK in *Clostridium perfringens*. J Bacteriol 191:2728–2742. doi:10.1128/JB.01839-08. 19201796 PMC2668419

[29] Smedley JG, III, McClane BA. 2004. Fine mapping of the N-terminal cytotoxicity region of *Clostridium perfringens* enterotoxin by site-directed mutagenesis. Infect Immun 72:6914–6923. doi:10.1128/IAI.72.12.6914-6923.2004. 15557612 PMC529159

[30] Shimizu T, Ohtani K, Hirakawa H, Ohshima K, Yamashita A, Shiba T, Ogasawara N, Hattori M, Kuhara S, Hayashi H. 2002. Complete genome sequence of *Clostridium perfringens*, an anaerobic flesh-eater. Proc Natl Acad Sci U S A 99:996–1001. doi:10.1073/pnas.022493799. 11792842 PMC117419

[31] Li J, Freedman JC, Evans DR, McClane BA. 2017. CodY promotes sporulation and enterotoxin production by *Clostridium perfringens* type A strain SM101. Infect Immun 85:e00855-16. doi:10.1128/IAI.00855-16. 28052992 PMC5328492

[32] Mi E, Li J, McClane BA. 2018. NanR regulates sporulation and enterotoxin production by *Clostridium perfringens* type F strain F4969. Infect Immun 86:e00416-18. doi:10.1128/IAI.00416-18. 30082481 PMC6204728

[33] Li J, Sayeed S, Robertson S, Chen J, McClane BA. 2011. Sialidases affect the host cell adherence and epsilon toxin-induced cytotoxicity of *Clostridium perfringens* type D strain CN3718. PLoS Pathog 7:e1002429. doi:10.1371/journal.ppat.1002429. 22174687 PMC3234242

[34] Li J, McClane BA. 2014. The sialidases of *Clostridium perfringens* type D strain CN3718 differ in their properties and sensitivities to inhibitors. Appl Environ Microbiol 80:1701–1709. doi:10.1128/AEM.03440-13. 24375134 PMC3957610

[35] Collie RE, Kokai-Kun JF, McClane BA. 1998. Phenotypic characterization of enterotoxigenic *Clostridium perfringens* isolates from non-foodborne human gastrointestinal diseases. Anaerobe 4:69–79. doi:10.1006/anae.1998.0152. 16887625

[36] Smedley JG, III, Saputo J, Parker JC, Fernandez-Miyakawa ME, Robertson SL, McClane BA, Uzal FA. 2008. Noncytotoxic *Clostridium perfringens* enterotoxin (CPE) variants localize CPE intestinal binding and demonstrate a relationship between CPE-induced cytotoxicity and enterotoxicity. Infect Immun 76:3793–3800. doi:10.1128/IAI.00460-08. 18505809 PMC2493238

[37] Kokai-Kun JF, Songer JG, Czeczulin JR, Chen F, McClane BA. 1994. Comparison of Western immunoblots and gene detection assays for identification of potentially enterotoxigenic isolates of *Clostridium perfringens*. J Clin Microbiol 32:2533–2539. doi:10.1128/JCM.32.10.2533-2539.1994. 7814493 PMC264097

[38] Bannam TL, Rood JI. 1993. *Clostridium perfringens*-*Escherichia coli* shuttle vectors that carry single antibiotic resistance determinants. Plasmid 29:233–235. doi:10.1006/plas.1993.1025. 8356117

[39] Li J, Freedman JC, McClane BA. 2015. NanI sialidase, CcpA, and CodY work together to regulate epsilon toxin production by *Clostridium perfringens* type D strain CN3718. J Bacteriol 197:3339–3353. doi:10.1128/JB.00349-15. 26260460 PMC4573732

[40] Freedman JC, Hendricks MR, McClane BA. 2017. The potential therapeutic agent mepacrine protects Caco-2 cells against *Clostridium perfringens* enterotoxin action. mSphere 2:e00352-17. doi:10.1128/mSphere.00352-17. PMC5577654 28875177

[41] Ma M, Gurjar A, Theoret JR, Garcia JP, Beingesser J, Freedman JC, Fisher DJ, McClane BA, Uzal FA. 2014. Synergistic effects of *Clostridium perfringens* enterotoxin and beta toxin in rabbit small intestinal loops. Infect Immun 82:2958–2970. doi:10.1128/IAI.01848-14. 24778117 PMC4097624

[42] Li J, Ma M, Sarker MR, McClane BA. 2013. CodY is a global regulator of virulence-associated properties for *Clostridium perfringens* type D strain CN3718. mBio 4:e00770-13. doi:10.1128/mBio.00770-13. 24105766 PMC3791898

[43] Zhao Y, Melville SB. 1998. Identification and characterization of sporulation-dependent promoters upstream of the enterotoxin gene (*cpe*) of *Clostridium perfringens*. J Bacteriol 180:136–142. doi:10.1128/JB.180.1.136-142.1998. 9422603 PMC106859

[44] Li J, McClane BA. 2010. Evaluating the involvement of alternative sigma factors SigF and SigG in *Clostridium perfringens* sporulation and enterotoxin synthesis. Infect Immun 78:4286–4293. doi:10.1128/IAI.00528-10. 20643850 PMC2950359

