# Supplementary figures and images for: NanH Is Produced by Sporulating Cultures of Clostridium perfringens Type F Food Poisoning Strains and Enhances the Cytotoxicity of C. perfringens Enterotoxin
Source: mSphere. 2021 Apr 28;6(2):e00176-21. doi: 10.1128/mSphere.00176-21 (PMC8092135; doi:10.1128/mSphere.00176-21)

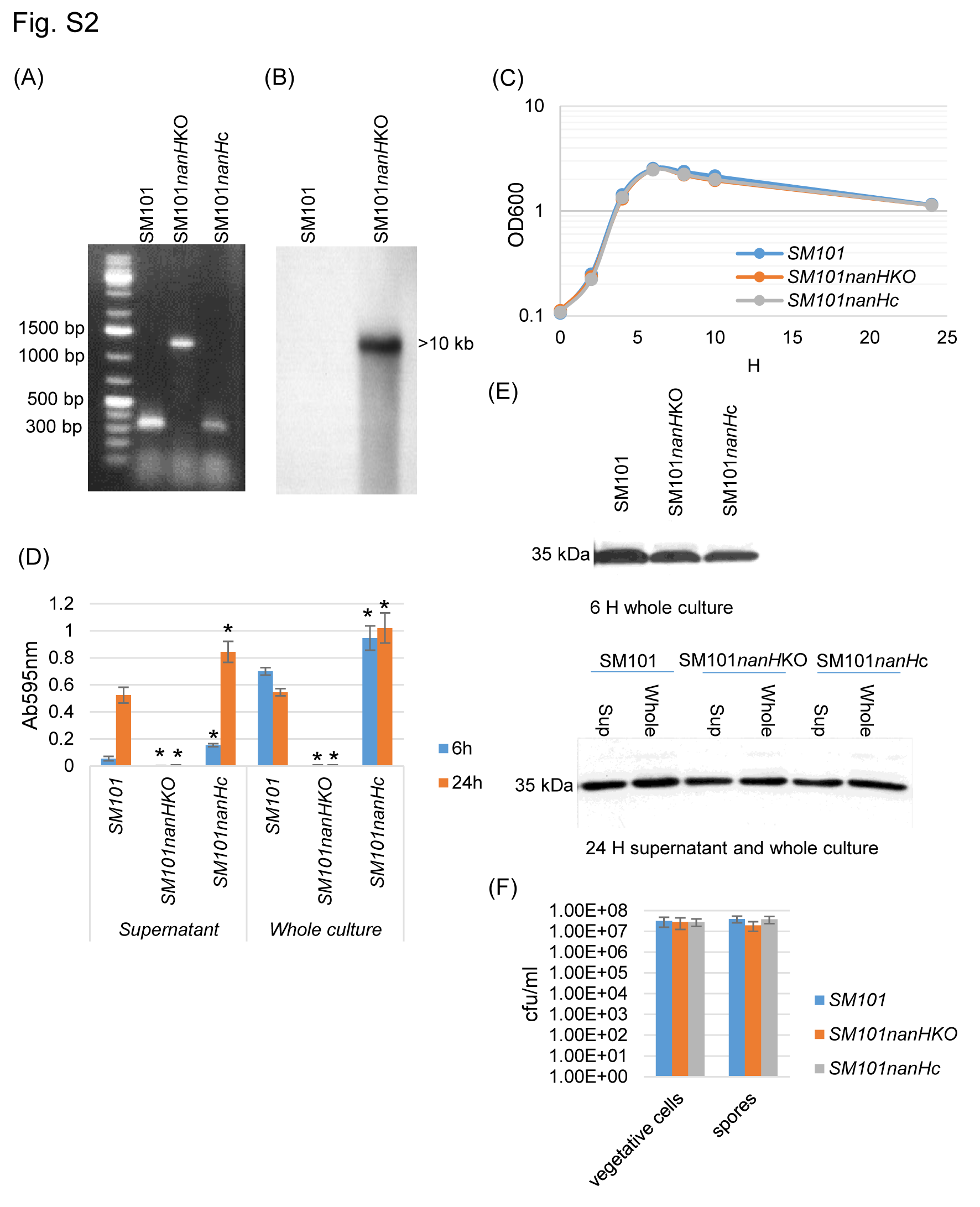

Supplement: FIG S2 [file mSphere.00176-21-sf002.tif]

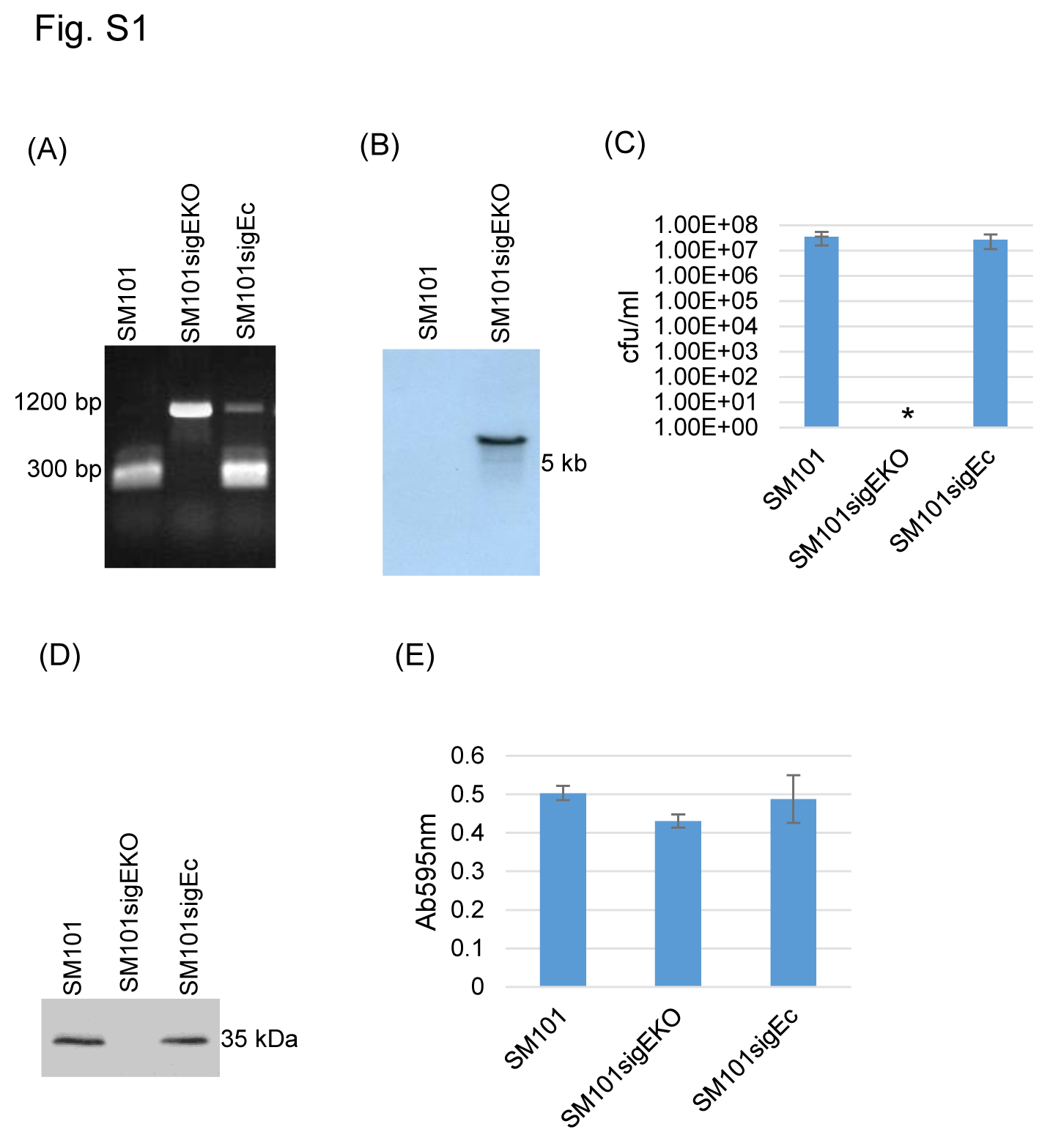

Supplement: FIG S1 [file mSphere.00176-21-sf001.tif]

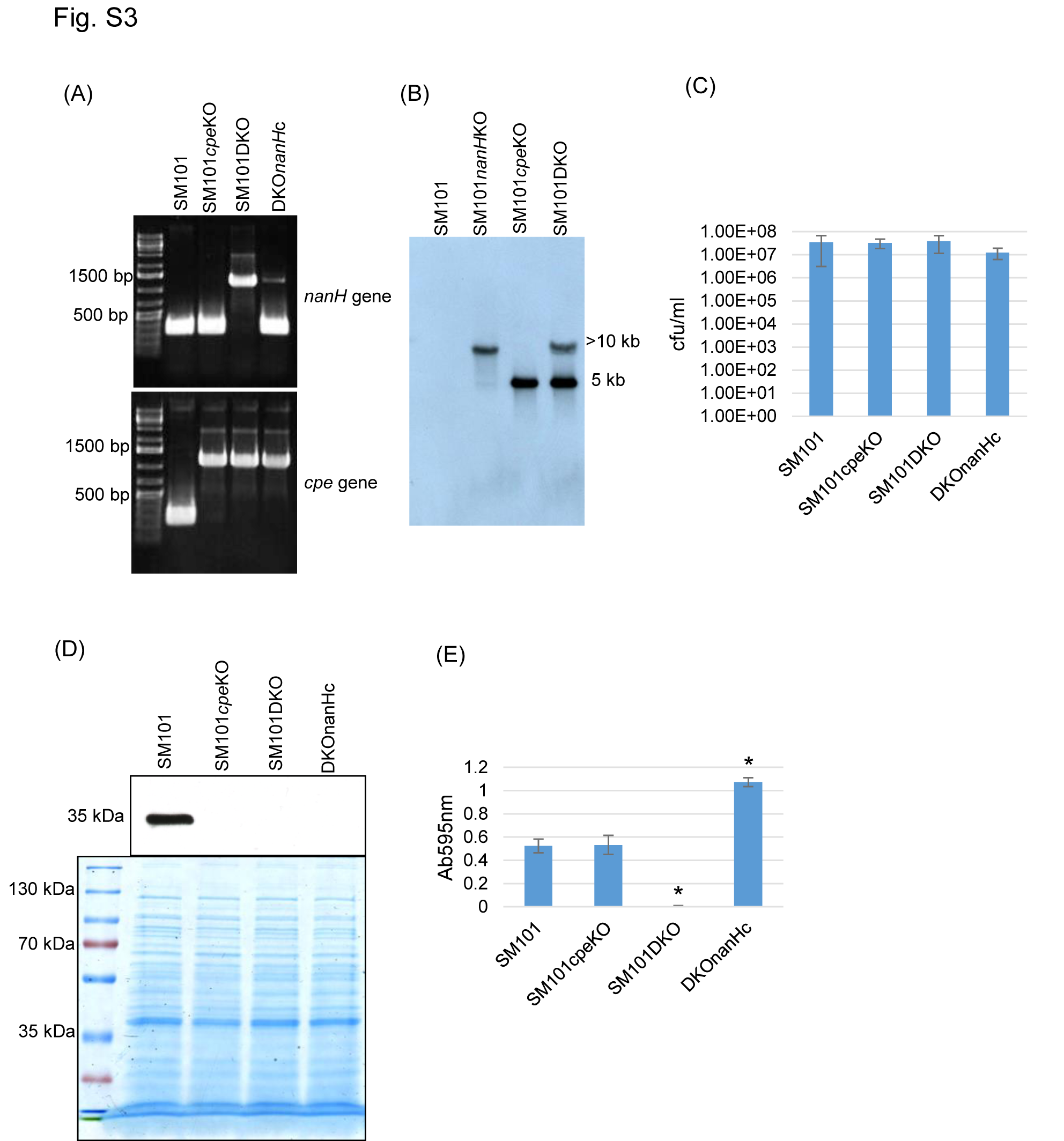

Supplement: FIG S3 [file mSphere.00176-21-sf003.tif]

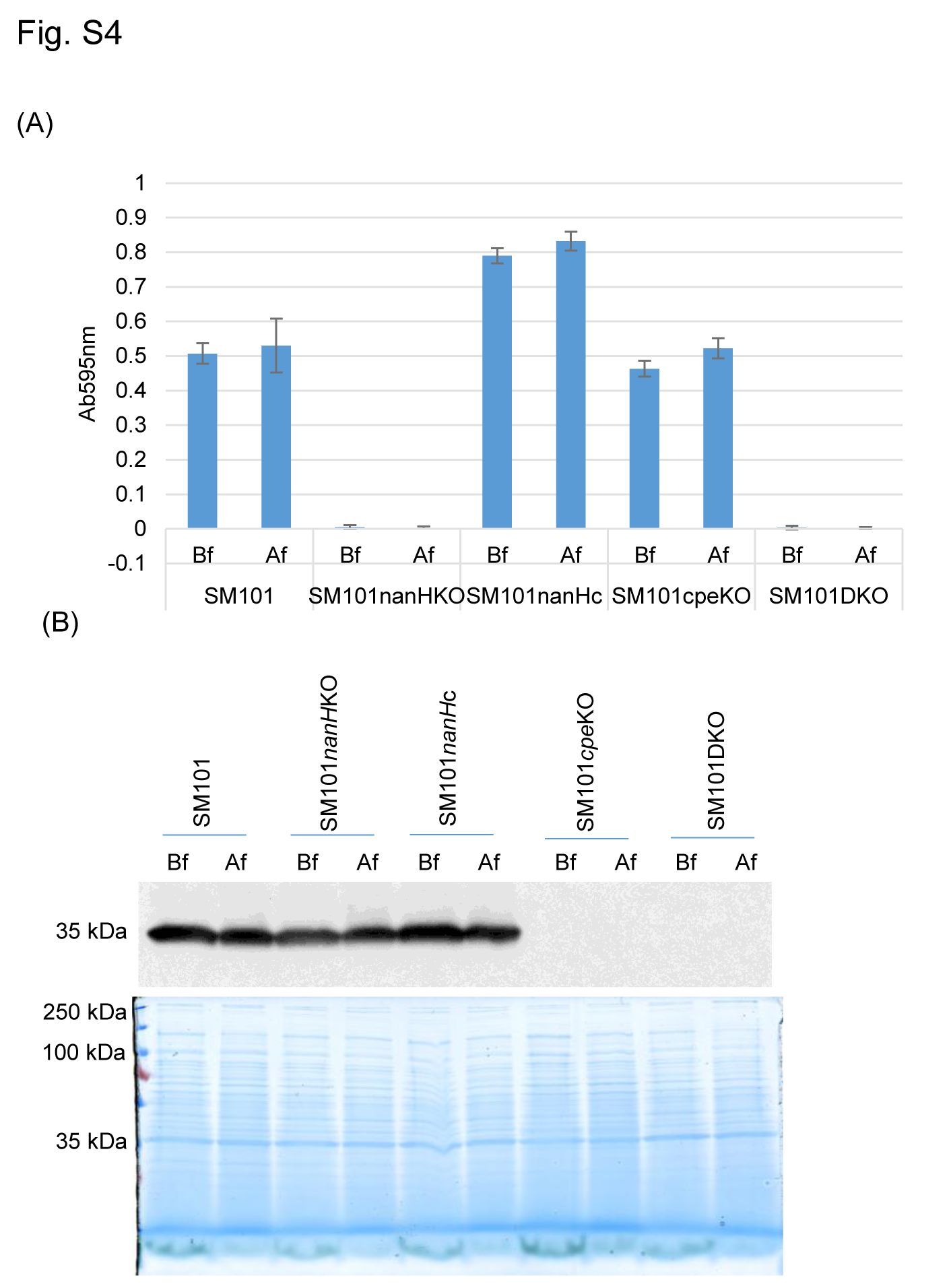

Supplement: FIG S4 [file mSphere.00176-21-sf004.tif]

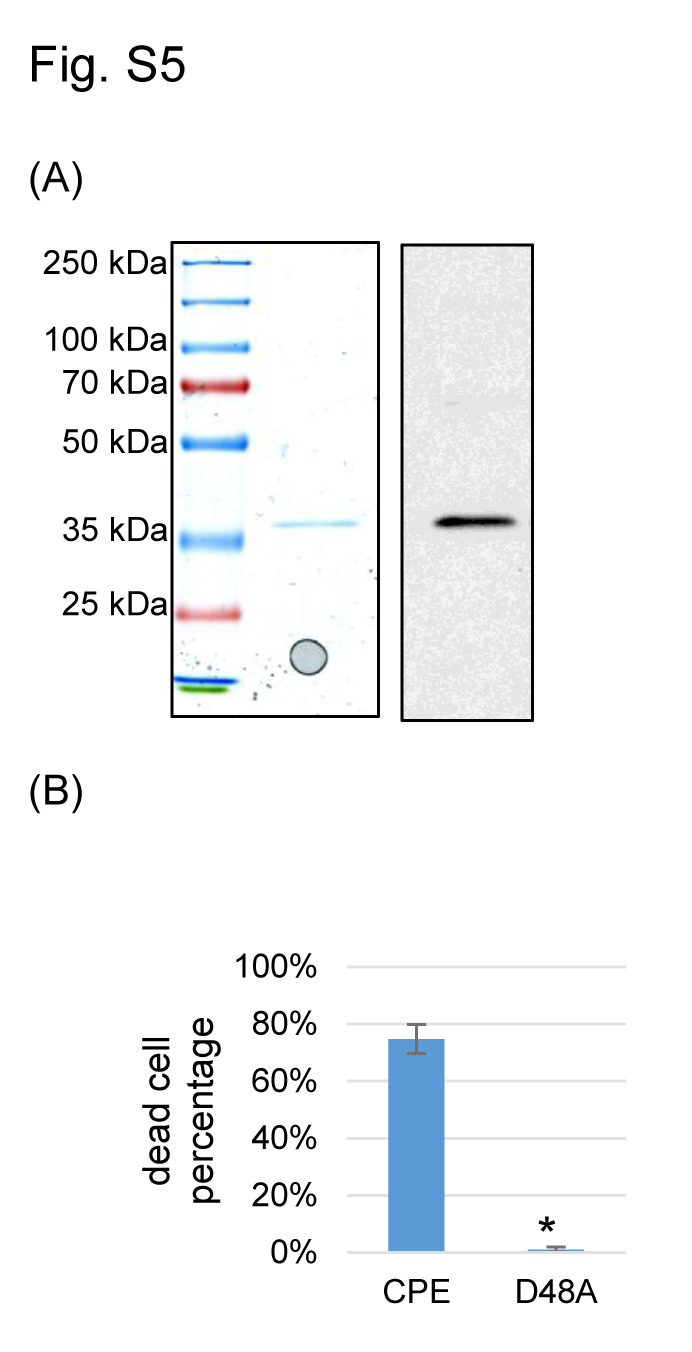

Supplement: FIG S5 [file mSphere.00176-21-sf005.tif]

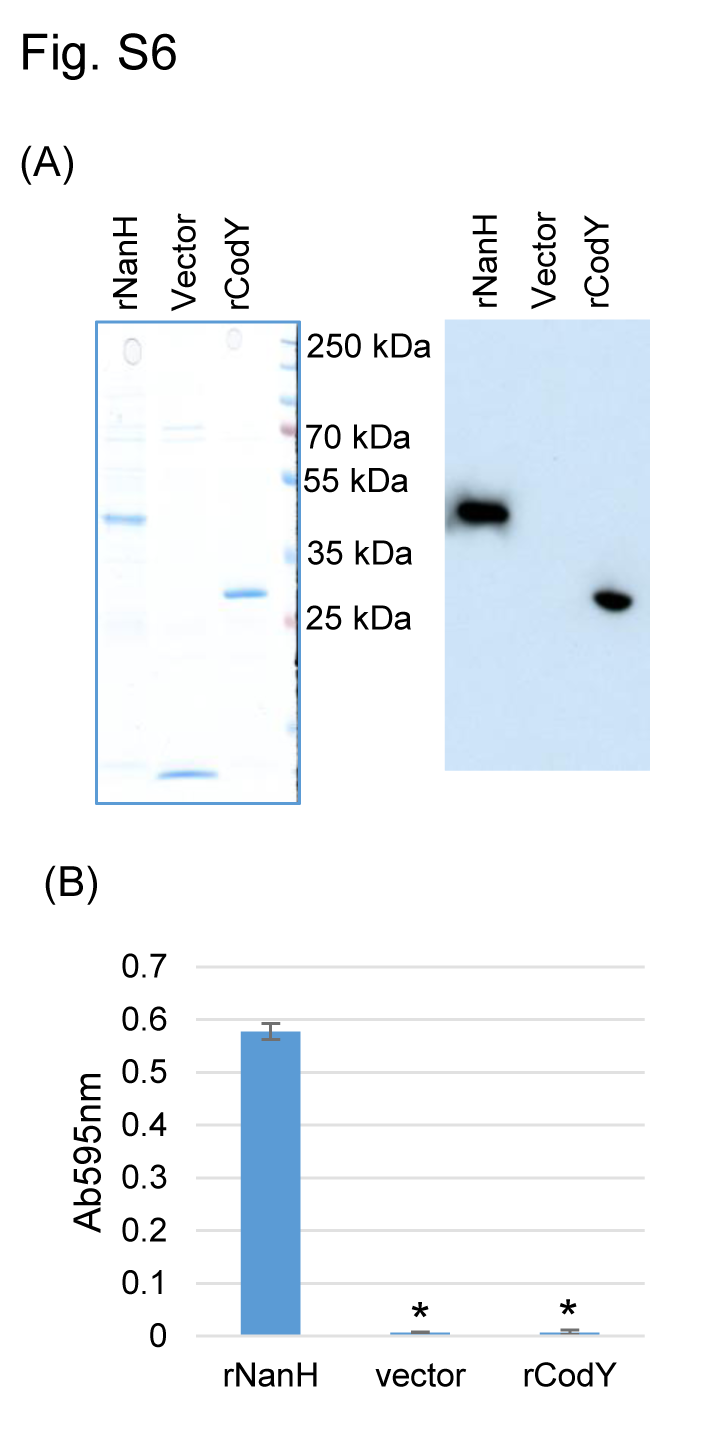

Supplement: FIG S6 [file mSphere.00176-21-sf006.tif]
